# Two-Phase Fermentation Systems for Microbial Production of Plant-Derived Terpenes

**DOI:** 10.3390/molecules29051127

**Published:** 2024-03-02

**Authors:** Tuo Li, Ximeng Liu, Haoyu Xiang, Hehua Zhu, Xuan Lu, Baomin Feng

**Affiliations:** College of Life and Health, Dalian University, Dalian 116622, China; liuximeng@s.dlu.edu.cn (X.L.); xianghaoyuu@163.com (H.X.); zhuhehua1@yeah.net (H.Z.); luxuan_232@163.com (X.L.)

**Keywords:** two-phase fermentation, plant-derived terpenes, microbial cell factory, in situ extraction, biosynthesis, downstream processing

## Abstract

Microbial cell factories, renowned for their economic and environmental benefits, have emerged as a key trend in academic and industrial areas, particularly in the fermentation of natural compounds. Among these, plant-derived terpenes stand out as a significant class of bioactive natural products. The large-scale production of such terpenes, exemplified by artemisinic acid—a crucial precursor to artemisinin—is now feasible through microbial cell factories. In the fermentation of terpenes, two-phase fermentation technology has been widely applied due to its unique advantages. It facilitates in situ product extraction or adsorption, effectively mitigating the detrimental impact of product accumulation on microbial cells, thereby significantly bolstering the efficiency of microbial production of plant-derived terpenes. This paper reviews the latest developments in two-phase fermentation system applications, focusing on microbial fermentation of plant-derived terpenes. It also discusses the mechanisms influencing microbial biosynthesis of terpenes. Moreover, we introduce some new two-phase fermentation techniques, currently unexplored in terpene fermentation, with the aim of providing more thoughts and explorations on the future applications of two-phase fermentation technology. Lastly, we discuss several challenges in the industrial application of two-phase fermentation systems, especially in downstream processing.

## 1. Introduction

Terpenes (hydrocarbons) or terpenoids (oxygen-containing derivatives), a highly chemically diverse family of natural products predominantly found in plants, possess a wide range of potent biological activities, including antimicrobial, antitumor, antiviral, antioxidant, anti-inflammatory, analgesic, digestive, and immunomodulatory effects [1,2,3]. Consequently, they have extensive applications in the medical field, food, and cosmetics. Notably, two terpene-based drugs, Taxol^®^ (anticancer) and artemisinin (antimalarial), have achieved clinical acclaim [4,5]. Traditionally, terpenes have been sourced primarily through plant extraction. Nevertheless, this method is hampered by several factors, such as slow plant growth, geographical limitations, and environmental conditions, which ultimately lead to low purity and yield, inefficient processes, and elevated costs. For example, producing just 2 g of pure Taxol^®^ requires about four trees of Pacific yew, highlighting the difficulty in meeting the escalating market and medicinal needs [4]. Chemical synthesis, as an alternative, often involves harsh conditions and lacks regioselectivity and stereoselectivity, complicating the synthesis of certain terpenes [6,7].

Recently, employing microorganisms for biosynthesizing plant-derived terpenes (PDTs) has proven effective [8,9,10,11]. Microorganisms offer advantages like rapid growth, genetic manipulability, environmental friendliness, and cost-effectiveness, making them suitable for terpene production [11,12,13]. Significant progress in this field includes constructing and regulating synthetic pathways, modifying key enzymes, and optimizing fermentation processes [11,13,14,15]. For example, Sudha Shukal and colleagues [16] achieved a significant milestone by biosynthesizing amorphadiene, a precursor of artemisinin, in *Escherichia coli*, yielding 30 g/L.

Between 1992 and 2001, several reviews addressed two-phase fermentation (TPF) systems, also known as “partitioning bioreactors”, “two-phase partitioning bioreactors (TPPB)”, extractive fermentation, or “in situ product removal (ISPR)” [17,18,19]. These systems, incorporating solvents or solids into cultures, effectively isolate specific metabolites, finding applications in various fields, including environmental biotechnology [20], and the microbial production of plant secondary metabolites [21,22]. A notable example is the use of TPF in amorphadiene production, where Keasling’s team [23] demonstrated that the two-phase culture significantly enhances volatile terpene yield by 8.5 times, markedly advancing terpene biosynthesis, particularly for volatile compounds.

Recently, several improved methods utilizing TPF systems have been developed for the biosynthesis of PDTs. For example, the resin Amberlite-XAD4, replacing organic solvents, has been employed for the TPF of α-humulene [24]. Additionally, alternative organic solvents like isopropyl myristate or methyl oleate have been used instead of n-dodecane for the fermentation of amorphadiene [25]. However, only a few reviews focusing on TPF systems for the production of PDTs have been reported. This review aims to discuss the recent advancements in TPF concerning the production of PDTs through microbial fermentation, with an emphasis on different types of TPF systems, their advantages, applications, influencing factors, limitations, and the economic considerations in downstream processing. For clarity and simplicity in subsequent discussions, the term ‘terpenes’ was chosen to collectively refer to all terpenoids produced by plants.

## 2. Types of TPF Systems

**Figure 1:**
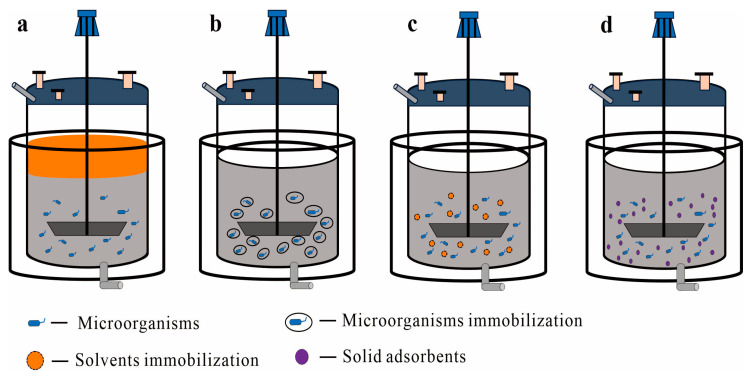
A concept of different types of TPF in a bioreactor: (**a**) liquid–liquid TPF systems, where the orange color on the upper layer represents organic solvents or another aqueous solvent and the dark gray color on the lower layer represents the culture medium; (**b**–**d**) different types of liquid–solid TPF systems: (**b**) immobilized cells as the SP; (**c**) immobilized solvent as the SP; (**d**) solid adsorbents as the SP.

The culture medium, known as the aqueous phase, supports cell growth, while the alternative phase, which can be liquid, solid, or a combination of both, is referred to as the SP. TPF systems are typically classified into liquid–liquid and liquid–solid systems based on the distinctive properties of the SPs [17,19,20,21] (Figure 1). This discussion aims to provide a comprehensive overview of TPF applications beyond PDT fermentation, highlighting diverse TPF systems’ potential across various fields. For instance, the integration of immobilized cells as a solid phase in terpene fermentation, despite its promise, remains underexplored.

### 2.1. Liquid–Liquid TPF Systems

Liquid–liquid TPF systems consist of two immiscible phases: an aqueous phase containing the microorganisms and nutrients, and an SP comprising a mixture of compounds, that may be water-insoluble, such as organic solvents or liquid lipophilic compounds, or water-soluble, such as polymers or salts [21,26,27] (Figure 1a). Systems with water-insoluble organic compounds are termed aqueous–organic two-phase systems (AOTPS), while those containing water-soluble compounds are referred to as aqueous two-phase systems (ATPS) [26,28].

#### 2.1.1. Aqueous-Organic TPF Systems

**Figure 2:**
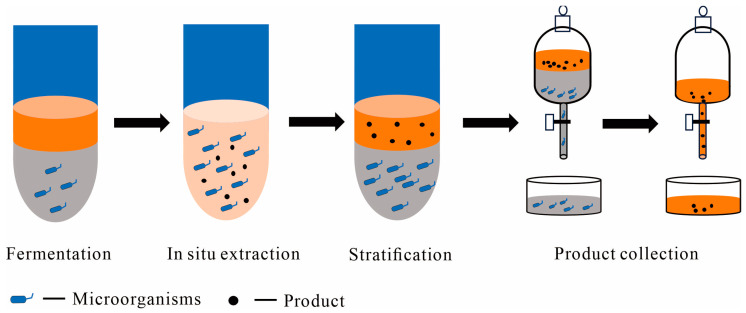
Scheme of aqueous–organic TPF and post-treatment, with main steps including fermentation, in situ extraction, post-fermentation stratification, and product collection; the orange color on the upper layer represents organic solvent or another aqueous solvent, the dark gray color on the lower layer represents the culture medium, and the light orange color of in situ extraction represents two phases mixed during the in situ extractive fermentation.

Aqueous-organic systems involve an aqueous and an immiscible organic phase, facilitating efficient product separation through in situ extraction [18,19,29]. The interaction between these phases allows for the dispersion of droplets, enhancing the extraction and separation of fermentation products [19,21] (Figure 2). Since Inoue and Horikoshi’s discovery in 1989 [30], which revealed varying tolerances of microorganisms to organic solvents and led to the isolation of the solvent-tolerant bacterium *Pseudomonas putida* IH 2000, organic solvents have been increasingly used in fermentation systems for in situ product extraction. For instance, Suzanne Verhoef and colleagues [31] utilized two solvent-tolerant *P. putida* S12 strains, employing glucose as the primary substrate to efficiently produce hydroxystyrene. This led to a final concentration of 21 mM, which was a fourfold increase compared to single-phase fed-batch cultivation. Similarly, Nicola Tan and colleagues [32] focused on trans-nerolidol, a valuable fragrance with antimalarial and anticancer properties, extensively used in cosmetics and agriculture. Under single-phase fed-batch fermentation, the strains produced over 6.8 g/L of nerolidol in 3 days. In contrast, two-phase extractive fed-batch fermentation yielded about 16 g/L of nerolidol in 4 days, with a carbon yield of approximately 9% (g/g), marking the highest yield achieved to date.

#### 2.1.2. Aqueous Two-Phase System

The aqueous two-phase system (ATPS), also referred to as an aqueous biphasic system (ABS), represents a biotechnological approach used in both fermentation and extraction processes [33,34]. It consists of two immiscible aqueous phases, usually formed by the combination of two water-soluble polymers, a polymer and a salt or two different salts. This system is environmentally preferable over traditional organic solvent-based TPF systems due to its aqueous nature and nonvolatile components [28,34]. The ATPS is primarily used in ex situ extraction processes, offering an alternative to conventional methods [28,35]. Ionic liquids (ILs), which are salts in the liquid state at low temperatures, have been effectively used for the extraction and purification of fermentation-derived components, showcasing sustainability, efficiency, and eco-friendliness [33,36]. However, the application of ATPSs for in situ extraction remains limited, mainly due to ATPSs’ cytotoxicity to cells and the challenge of finding an appropriate formulation for the fermentation process.

H. González-Peñas and coworkers [37] performed a solvent screening for in situ liquid extraction from acetone–butanol–ethanol (ABE) fermentation by *Clostridium acetobutylicum.* They selected methyltrioctylammonium chloride and trihexyl (tetradecyl) phosphonium chloride for their extraction capacity, demonstrating high distribution coefficients. Despite lower selectivity due to significant water extraction, this research highlighted ILs’ potential for in situ extraction processes. Deep eutectic solvents (DESs) represent an emerging class of eutectic mixtures of Lewis or Brønsted acids and bases, capable of forming a variety of anionic and cationic species [38,39]. Due to the similar characteristics and properties with ILs, DESs are widely known as IL analogs. For example, Liu Jingyang and his team [40] chose a DES composed of choline chloride and urea for the in situ extraction of L-valine produced by *Brevibacterium flavum* XV0505. Optimizing the timing and volumetric fraction of the IL addition, it was shown that adding 0.1% of this IL at the 16th hour of the fermentation process led to the XV0505 strain producing the highest yield of L-valine in both shake-flask and fed-batch fermentation experiments. Similarly, Parul Badhwar and colleagues [41] selected the *Aureobasidium pullulans* strain for cost-effective pullulan production and developed a new ATPS for fermentation. They conducted a comprehensive study of the effects of different molecular weights of polyethylene glycol (PEG) (400, 600, 4000, and 6000) and dextran or mono/di-sodium phosphate salts on the fermentation process. The PEG-dextran ATPS system was found to be suitable for the extractive fermentation of pullulan from *A. pullulans*, achieving a yield of 36.47 g/L. Although ILs showed lower selectivity, their high distribution coefficients indicate a strong potential for targeted extractions, highlighting the need for further optimization in selecting ILs for in situ fermentation processes [42].

### 2.2. Liquid–Solid TPF Systems

As defined by Sonia Malik et al. [21], liquid–solid TPF systems involve in situ adsorption with an aqueous medium and a solid phase comprising adsorbents or lipophilic materials. This review expands on the use of solids as an SP, including the integration of immobilized cells or solutions as the solid phase [43,44] (Figure 1b–d). Immobilization refers to the containment or fixation of cells or solutions on or within a matrix [44,45,46]. This process prevents their release during the fermentation while ensuring adequate permeability for the diffusion of substrates and products [45]. In this context, the immobilized cells or solutions effectively serve as a solid phase (Figure 1b,c). Liquid–solid systems show distinct advantages, such as simplified post-fermentation processing and the reusability of immobilized cells.

#### 2.2.1. Immobilized Cells as the Solid Phase

**Figure 3:**
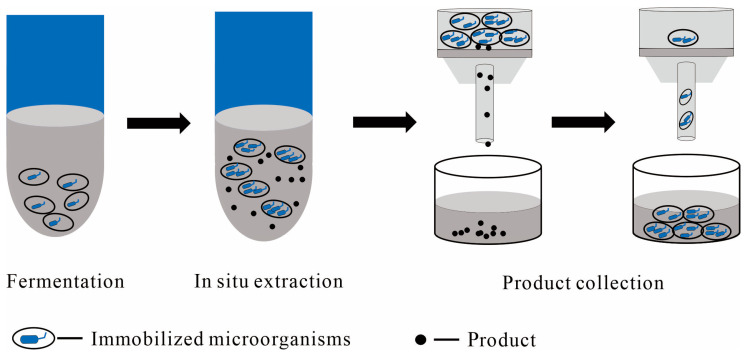
Illustration of a flowchart using immobilized cells as the solid phase in the liquid–solid TPF and downstream processing, with main steps including fermentation, in situ extraction, post-fermentation filtration, and product collection; the dark gray color represents the culture medium.

Immobilized cells involve anchoring active cells (as shown in Figure 3), serving as biological catalysts, onto a carrier to create a stable structure [43]. Key carriers include solid particles, gels, and membranes [46]. This approach improves cell stability and reusability, thereby streamlining operational and control processes and enhancing their suitability for industrial applications.

Cell immobilization techniques encompass a variety of methods [43,47]. Adhesion and adsorptive immobilization entail the physical adsorption of cells onto a carrier’s surface. This process depends on the physicochemical properties of both the cells and the carrier. For instance, brewing yeast immobilization onto spent grains involves cell–carrier adhesion, cell–cell attachment, and cell adsorption inside carrier crevices, affected by factors like dilution rate and the hydrophobicity of the carrier [48]. Covalent bonding immobilization involves attaching cells to a carrier via covalent bonds. The efficiency of immobilization is significantly influenced by the quantity and accessibility of reactive groups on the cell surface, which are in turn affected by physiological factors. Thus, covalent coupling is not a major technique used for cell immobilization [47]. Encapsulation immobilization, also known as microencapsulation, involves encapsulating cells within a carrier to create anchored colloidal particles. This technique encompasses coating or entrapping microbial cells with a polymeric material, resulting in the formation of microspheres [49]. Microencapsulation provides several benefits, including increased cell loading capacity, improved cell survival, and a higher production rate of desired microbial products [50]. This technology has been widely applied in various fields involving microbial cells, including the microencapsulation of probiotics [51]. Gel immobilization is characterized by immobilizing cells within a gel-like substance, while entrapment immobilization traps cells within a matrix or polymer. The design of robust matrices, such as macroporous gels with immobilized microbial cells, has shown high efficiency and structural stability [46]. These gels allow for the high retained activity of yeast and *E. coli* cells even after drying and storage, demonstrating their practicality in stirred bioreactors [43,46].

Immobilized cells or enzymes are widely used in biotransformation, with recent papers offering comprehensive overviews of cell or enzyme immobilization techniques in this field [43,46,47]. However, the utilization of immobilized cell technology for producing PDTs remains limited. For example, El-Sayed R. et al. [52] immobilized two mutant strains of *Aspergillus fumigatus* and *Alternaria tenuissima* using five different entrapment carriers of calcium alginate, agar-agar, Na-CMC, gelatin, and arabic gum. Among these, calcium alginate gel beads proved to be the most effective and suitable entrapment carrier for maximum production of paclitaxel. Considering the limited cell immobilization reports, the present part aims to introduce several representative examples involving fermentative production via immobilized cell technology, with the goal of providing useful references and insights for the production of PDTs.

Product inhibition by butanol and acetone is a significant limitation in ABE fermentation. Rizki Fitria Darmayanti and colleagues [53] developed an innovative biobutanol extractive fermentation method using a large volume ratio of extractant with immobilized *Clostridium saccharoperbutylacetonicum* N1-4. The preculture cells of the N1-4 strain were fixed in calcium alginate beads, effectively maintaining a low butanol concentration in the aqueous phase and achieving a total butanol concentration of 64.6 g/L. In a study conducted by Sion Ham and colleagues [54], they utilized engineering techniques and immobilized whole cells of *E. coli* to establish a small-scale reactor system, successfully achieving continuous and efficient production of γ-aminobutyric acid (GABA). Remarkably, these anchored cells maintained high activity after 15 consecutive uses, whereas free cells lost activity after the ninth reaction. Moreover, after optimizing conditions such as buffer concentration, substrate concentration, and flow rate, the researchers successfully achieved continuous operation for 96 h in a 14 mL scale reactor, producing a total of 165 g of GABA. This research not only presents a viable method for producing high concentrations of GABA but also highlights the superior performance of immobilized microbial cells in the process. Weysser Felipe Cândido de Souza et al. [55] utilized an immobilization system comprising 2.0% *w*/*v* alginate, 2.0% *w*/*v* CaCl_2_, 2.0% *w*/*v* gelatin, and 0.2% *w*/*v* transglutaminase to immobilize *Erwinia* sp. D12 cells. Their experiments revealed that isomaltulose production reached its peak at 327.83 g/L within the first 24 h and that the cells remained stable over 72 h of continuous reaction, maintaining consistent isomaltulose output. This demonstrates that using ionic gelation to immobilize *Erwinia* sp. D12 cells are an effective method for enhancing sucrose-to-isomaltulose conversion. *S. cerevisiae*, a widely favored chassis cell, has shown tremendous potential in producing PDTs [56,57]. Although the technology for immobilizing *S. cerevisiae* cells has not yet been applied in the aforementioned fermentation field, the techniques for immobilizing or encapsulating *S. cerevisiae* cells are already quite mature in other biotransformation areas. These studies provide valuable experience and reference for future use of immobilized yeast in the production of terpenoid compounds.

#### 2.2.2. Immobilized Solvent as the Solid Phase

**Figure 4:**
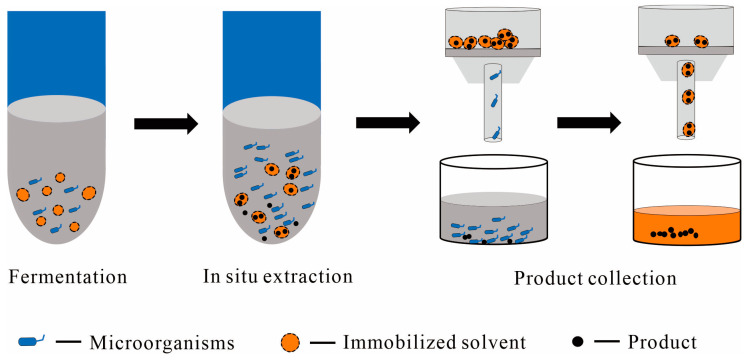
A conceptual scheme of using immobilized solvent as the solid phase in the liquid–solid TPF and downstream processing, with main steps including fermentation, in situ extraction, post-fermentation filtration, and product collection; the dark gray color represents the culture medium and the orange color represents solvents, like ILs.

The solution immobilization system involves integrating solution chemical substances with solid carriers to create solid particles or agglomerates, as illustrated in Figure 4. This technology is commonly used in separation and purification processes, enhancing product purity and minimizing waste. The previous discussion of liquid–liquid TPF introduced several biphasic systems, with a focus on the immobilization of ILs [44,58]. ILs are commonly immobilized onto materials like silica or polymers through physical confinement or covalent grafting, mainly enhancing organic catalysis and separation.

For example, Changhee Lee and colleagues [59] immobilized the lipase B (CALB) from *Candida Antarctica* and 1-octyl-3-methylimidazolium tetrafluoroborate in a polymeric hybrid monolith, obtaining an enzyme-SILP (e-SILP) catalyst. This catalyst was effective in continuous gas-phase transesterification of vinyl propionate and 2-propanol. Additionally, ILs were anchored on silica as a stationary phase for compound separation and purification. Another application of solution immobilization involved the same CALB in ester enzyme reaction systems. To address the solubility mismatch between enzymes and substrates, a Pickering gel emulsion stabilized by enzyme-modified polymer nanomaterials was developed, facilitating biphasic biocatalysis. These nanomaterials, produced surfactant-free via emulsion polymerization and covalently attached to CALB, were mixed with heptane to create an aqueous dispersion, enhancing nanoparticle decoration. Impressively, CALB immobilized in this emulsion achieved a 96.5% conversion rate and retained 92.5% of its activity after 10 reaction cycles [60]. Similarly, Susanne Wiese et al. [61] employed microgels in emulsions to improve the interaction between oil and water phases, forming droplets encapsulating both enzyme- and substrate-containing oil. The microgels positioned at the droplet interface facilitated substrate conversion. Post-reaction, heating beyond the microgels’ volume phase transition temperature induced emulsion breakdown, which allowed for product recovery via macroscopic phase separation.

#### 2.2.3. Solid Adsorbents as the Solid Phase

**Figure 5:**
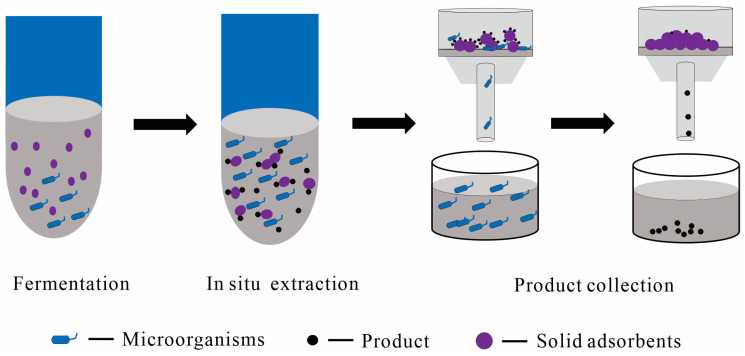
A conceptual flowchart using solid adsorbents as the solid phase in the liquid–solid TPF and downstream processing, with main steps including fermentation, in situ extraction, post-fermentation filtration, and product collection; the dark gray color represents the culture medium.

Solid adsorbents like polymer beads and resins are preferred for in situ extractive fermentation due to their ability to efficiently adsorb and remove products from the aqueous phase, simplifying the process by eliminating extra separation steps (Figure 5). These adsorbents, particularly effective for volatile compounds, offer a nontoxic alternative to organic solvents.

As early as 2009, Guillermo Quijano et al. [20] detailed the use of solid-phase adsorbents in TPF for environmental biotechnology, notably in wastewater treatment. Sonia Malik et al. [21] provided insights into the application of adsorbent resins in plant cell fermentation, with a dedicated chapter focusing on the selection of appropriate adsorbents and operating conditions. Furthermore, Thomas Phillips and colleagues [22] explored the use of adsorbent resins in the microbial fermentation of natural products, employing in situ solid-phase adsorption techniques. Their paper not only delved into the underlying mechanisms but also examined the influence of in situ adsorption on the biosynthesis of microbial natural products. Given this extensive precedence, our objective here is to provide a concise overview of this technology, highlighting notable examples. The application of adsorbent resins in microbial fermentation systems for the production of PDTs will be discussed in detail in a subsequent section.

Jianxu Li and coworkers [62] evaluated an integrated in situ fermentation and in situ product recovery process aimed at enhancing the output of the antibiotic compound beauvericin (BEA) in *Fusarium redolens* Dzf2 mycelial cultivation. For this purpose, they employed macroporous polystyrene resin (X-5) as the adsorbent (encased in nylon bags), introducing it into flasks containing fungal mycelia. The findings indicated a significant increase in BEA volumetric production, from 194 mg/L to 265 mg/L by Day 7, with 65% of BEA adsorbed onto the resin. Renewing the resin and adding glucose on Day 7 further elevated BEA output to 400 mg/L by Day 9, effectively doubling the yield compared to the batch control culture. Haishan Qi et al. [63] introduced adsorbent resin HP20 during the fermentation of *Streptomyces hygroscopicus* var. *ascomyceticus* FS35. Following a metabolic profiling analysis and subsequent rational fermentation optimization, the production of ascomycin by *S. hygroscopicus* var. *ascomyceticus* FS35 significantly increased to 460 mg/L in a 168 h fermentation period. This represents a 53.3% enhancement compared to the yield under initial fermentation conditions. These case studies highlight the potential of solid adsorbent strategies, particularly adsorbent resins, in amplifying the production of significant natural products and refining processes.

## 3. The Advantages of TPF Systems

In microbial fermentation, increased yield is often hindered by the accumulation of fermentation products. Integrating fermentation with in situ extraction presents an effective strategy to mitigate this issue. This integrated approach accelerates product formation, boosts yield, and simplifies downstream processing. Among various two-phase systems, aqueous–organic and liquid–solid (resin) TPF technologies are particularly prominent and mature in microbial fermentation. Thus, subsequent chapters will extensively discuss the benefits of these TPF systems.

### 3.1. Enhance Productivity

Numerous studies have demonstrated that in situ product extraction, employing either a liquid (organic solvent) or solid (resin) phase, significantly enhances production. Microbial cells in the culture medium synthesize products, which are then extracted or adsorbed by the SP, disrupting the equilibrium and promoting product release. For instance, in β-elemene biosynthesis by *E. coli*, strategies like efflux protein enhancement and the use of n-dodecane as an organic phase in fermentation increased the β-elemene yield to 3.52 g/L [64].

#### 3.1.1. Reducing Toxicity to Microbial Cells

Targeted products and harmful metabolites released during fermentation can inhibit microbial growth and production. Some monoterpenes and phenolic compounds can impair cell walls, membranes, and organelle membranes, diminish the activity of specific enzymes within the cells, obstruct normal cellular functions, and ultimately result in microbial death. TPF technology, by enabling simultaneous production and separation, efficiently extracts or adsorbs both products and nontarget metabolites, enhancing microbial tolerance and productivity. For example, Wei Liu and colleagues [65] discovered that during batch-fed fermentation of an engineered *E. coli* strain producing geraniol, introducing isopropyl myristate to establish an aqueous–organic two-phase system significantly prevented the volatilization of the target product and diminished its cellular toxicity. This method resulted in a notable increase in product yield.

#### 3.1.2. Alleviating Feedback Inhibition

Product accumulation may lead to feedback inhibition, impeding the activity of enzymes in the biosynthetic pathway. While most secondary metabolites produced by microbial cells are hydrophobic with low solubility in the culture medium, even minimal concentrations can inhibit enzymes involved in their biosynthesis. TPF is instrumental in facilitating in situ extraction or adsorption of products, effectively alleviating feedback inhibition in the biosynthetic pathway or affecting cell membrane transport. For example, in the study conducted by Jorge H. Santoyo-Garcia et al. [66], it was found that the resin could remove the products/reactive oxygen species’ (ROS) effects in the production of paclitaxel by *Taxus baccata* vascular stem cells. This removal is crucial as it prevents the activation of secondary undesired pathways, inhibits cell growth, or diverts the metabolic flux towards side products.

#### 3.1.3. Preventing Product Degradation and Loss

In the fermentation process, some enzymes in microbial cells or acidic substances in the fermentation system can degrade certain metabolites, particularly at high concentrations. The TPF systems can ensure that secondary metabolites are maximally protected from degradation by the microorganisms’ own enzymes, effectively limiting the loss of products in cell culture. Taking salinosporamide A as an example, this natural molecule is produced by marine actinomycete *Salinispora tropica* and has a half-life of 140 min. Adding 2% (*w*/*v*) XAD-7 resin at 24 h of fermentation increased the yield from 5.7 mg/L to 278 mg/L, suggesting that the resin may protect the product from degradation [67]. The hydrophobic and volatile characteristics of some terpenes primarily contribute to product loss in microbial production processes. To mitigate the volatile losses of terpenes, a prevalent strategy is employing TPF with organic solvents. These solvents not only decrease the volatility of terpenes but also reduce their toxicity to cells, thereby enhancing productivity.

### 3.2. Industrial Application: Cost-Effective and Downstream Processing

The primary goal of microbial fermentation research is scaling to industrial production, often hindered by complex and costly downstream processes. TPF technology can address this challenge, reducing post-fermentation costs and facilitating scale-up.

#### 3.2.1. Increase in Cell Biomass and Recycling of the Second Phase

In TPF systems, the SP extracts or adsorbs cellular products, fostering cell growth and increasing microbial cell biomass and yield compared to traditional approaches. On the other hand, the recycling of the SP is another key feature of the TPF system. In industrial production, effective separation and recycling techniques allow multiple uses of the SP, minimizing downstream processing costs.

#### 3.2.2. Reduction of Post-Processing Steps

Utilizing TPF systems obviates the need for intricate product harvesting procedures, preserving cell integrity and not interfering with the culture process, thereby minimizing the costs and time. Traditional fermentation typically necessitates numerous steps such as organic solvent extraction, concentration, and distillation to isolate the product. TPF technology streamlines these processes. For instance, in liquid–liquid TPF, the product, extracted by the SP organic solvent during fermentation, eliminates the need for extraction. The fermentation broth is centrifuged, and the organic solvent is directly concentrated, followed by distillation to retrieve the product. In solid–liquid TPF, a concentration step is unnecessary; organic solvents are directly employed to elute and extract from adsorbents like macroporous resins, followed by distillation, reducing industrial post-processing steps.

## 4. Applications of TPF in Microbial Production of Terpenes

Terpenes represent a very important class of secondary metabolites in plants, with over 80,000 structural types identified to date [68]. These compounds, composed of isoprene units (C5 units), vary in the number of isoprene units they contain [69], leading to classifications such as monoterpenes (C10), sesquiterpenes (C15), diterpenes (C20), sesterterpenes (C25), triterpenes (C30), sesquarterpenes (C35), tetraterpenes (C40), and polyterpenes (C > 40). Currently, there are several marketed plant-derived terpenoid drugs, such as paclitaxel, β-elemene, and artemisinin. Additionally, terpenes are highly favored in the fragrance and cosmetic industry, featuring components like menthol and ambergris [70].

**Figure 6:**
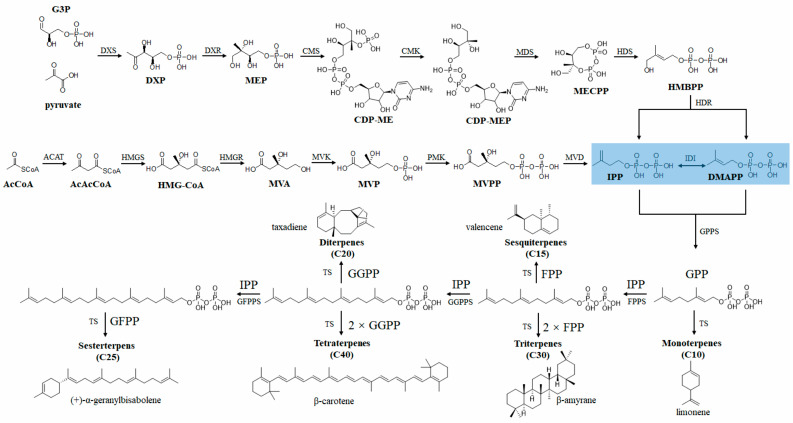
Metabolic pathway of terpene biosynthesis. The terpenes’ skeletons are formed by the condensation of multiple units of IPP and its isomer, DMAPP. MEP biosynthetic pathway starts with pyruvate and G3P. Through a series of enzyme-catalyzed reactions, it ultimately produces IPP and DMAPP (shown on a light blue background). This process involves a variety of enzymes, DXS (1-deoxy-d-xylulose-5-phosphate synthase) and DXR (1-deoxy-d-xylulose-5-phosphate reductoisomerase), CMS (2-C-methyl-d-erythritol 4-phosphate cytidyltransferase), CMK (4-diphosphocytidyl-2-C-methyl-d-erythritol kinase), MDS (2-C-methyl-d-erythritol 2,4-cyclodiphosphate synthase), HDS (4-hydroxy-3-methylbut-2-enyl diphosphate synthase), and HDR (4-hydroxy-3-methylbut-2-enyl diphosphate reductase). The MVA biosynthetic pathway, which is another pathway for terpene biosynthesis, distinct from the MEP pathway, starts with acetyl-CoA. The primary enzymes involved in the MVA pathway are acetyl-CoA acetyltransferase (ACAT), hydroxymethylglutaryl-CoA synthase (HMGS), hydroxymethylglutaryl-CoA reductase (HMGR), mevalonate kinase (MVK), phosphomevalonate kinase (PMK), mevalonate-5-pyrophosphate decarboxylase (MVD), and isopentenyl-diphosphate delta-isomerase (IDI). Geranyl diphosphate synthase (GPPS), farnesyl diphosphate synthase (FPPS), geranylgeranyl diphosphate synthase (GGPPS), and farnesylgeranyl diphosphate synthase (GFPPS) TPs convert the basic terpene precursors, IPP and DMAPP, into various terpene compounds. Abbreviation of metabolites: DXP, 1-deoxy-d-xylulose-5-phosphate; MEP, 2-C-methyl-d-erythritol-4-phosphate; CDP-ME, 4-diphosphocytidyl-2-C-methyl-d-erythritol; CDP-MEP, 4-diphosphocytidyl-2-C-methyl-d-erythritol 2-phosphate; MEcPP, 2-C-methyl-d-erythritol-2,4-cyclodiphosphate; HMB-PP, 4-hydroxy-3-methylbut-2-enyl-diphosphate; AcAc-CoA, acetoacetyl-CoA; HMG-CoA, 3-hydroxy-3-methylglutaryl-CoA; MVA, mevalonate; MVP, mevalonate-5-phosphate; MVPP, mevalonate-5-pyrophosphate; IPP, isopentenyl pyrophosphate; DMAPP, dimethylallyl pyrophosphate; GPP, geranyl pyrophosphate; FPP, farnesyl pyrophosphate; GGPP, geranylgeranyl diphosphate; GFPP, farnesylgeranyl diphosphate.

The biosynthesis of terpenes in plants is complex but well understood [8,10,11,70]. As shown in Figure 6, isoprene isopentenyl diphosphate (IPP) and dimethyl allyl phosphate (DMAPP) are common precursors for all terpenes. One molecule of DMAPP and varying numbers of IPP condense under the influence of prenyltransferases to produce different terpene precursors. These precursors are then converted into various terpene skeletons under the action of various terpene synthases (TPs). The mevalonate (MVA) and 2-C-methyl-d-erythritol 4-phosphate (MEP) pathways are two distinct metabolic routes for the biosynthesis of terpenes in plants. The MVA pathway starts with acetyl-CoA and proceeds through six enzymatic steps to produce IPP and DMAPP, the basic building blocks for isoprenoid synthesis [71]. The MEP pathway uses pyruvate and glyceraldehyde 3-phosphate (G3P) as substrates and involves seven enzymatic reactions to produce IPP and DMAPP [72]. Given the well-characterized biosynthetic pathways of PDTs, the utilization of microbial engineering holds significant potential as an effective alternative for the production of desired terpenes.

### 4.1. Microbial Production of Plant-Derived Terpenes

Microorganisms with a short growth cycle and minimal environmental impact are an ideal choice for PDT biosynthesis [73]. The microbial synthesis of terpenes, particularly with *E. coli* and *S. cerevisiae*, aligns with the goals of green and sustainable development due to their well-characterized metabolic pathways, genetic tractability, and suitability for large-scale fermentation [14]. These organisms offer the benefits of operational simplicity and cost-effectiveness, leveraging inexpensive substrates for efficient growth [74]. Further, the advancement in molecular and synthetic biology has led to the successful utilization of other microbial chassis such as *Yarrowia lipolytica* and *Rhodosporidium toruloides* in terpenoid biosynthesis. *Y. lipolytica*, recognized for its lipid production and “Generally Recognized as Safe” (GRAS) status [75], excels at utilizing renewable carbon sources and exhibits a high acetyl-CoA flux, making it a potent producer of acetyl-CoA-derived products [76]. *R. toruloides*, known for its broad substrate range and inhibitor tolerance, emerges as another viable host for high-value compound production [77]. TPF technology, encompassing both aqueous–organic solvent and aqueous–solid TPF systems, is pivotal in the microbial production of terpenes, enhancing the efficiency and quality of products such as β-elemene [78] and artemisinic acid [79]. The modifications of terpenes’ biosynthetic pathways in the microbial cell factory and the key enzymes engineering this process have been discussed by other comprehensive reviews. In this part, we focus on terpenes with validated fermentation experiments, emphasizing the preferred mode of microbial fermentation production, and exclude those only studied for their biosynthetic pathways, such as sesquiterpenes and sesquarterpenes.

Given the scope of this review, we have comprehensively collated most of the results concerning plant-derived terpenes in the process of microbial fermentation up to November 2023, which can be found in Supplementary Materials Tables S1–S5. For illustrative purposes, we have selectively highlighted the top-yielding examples of each terpene achieved using various chassis cells, employing different fermentation techniques (such as two-phase and non-two-phase fermentation), and across diverse fermentation systems and scales.

#### 4.1.1. Monoterpenes

Monoterpenes, the simplest terpenes comprising two isoprene units, are key to the aromatic profiles of many plants’ essential oils [70]. Recent TPF applications have shown significant promise in enhancing monoterpene production, and Table 1 provides a comprehensive overview of these recent advancements. Taking geraniol as an example, it is not only an acyclic monoterpene isolated from plant essential oils that has been extensively utilized in the flavor industry for the past few decades but has also garnered considerable interest in recent years as a potential biofuel [65]. By overexpressing the synthase and heterologous MVA pathway in *E. coli* and subsequently employing fed-batch fermentation with isopropyl myristate as the organic phase, the production of geraniol was significantly enhanced, resulting in a yield of 2 g/L compared to the initial 78.8 mg/L obtained after basic fermentation in a bioreactor [65]. In another study, by adding the same SP in fed-batch fermentation, the production of geraniol was further increased to 13.19 g/L [80]. Different organic solvents, such as n-decane [81] and n-dodecane [82], had been employed as the SP in the TPF, which also increased the production of geraniol.

The demand for linalool, particularly as a flavoring agent, has been escalating, especially in the realm of processed foods and beverages. Achieving stable and cost-effective production of linalool is essential, as current extraction methods yield limited quantities and are not economically viable. To overcome the volatility of linalool in aqueous solutions and its high toxicity to microorganisms during fermentation production, an in situ extraction fermentation using isopropyl myristate as the SP was developed, resulting in 5.60 g/L (*S*)-linalool and 3.71 g/L (*R*)-linalool [113].

Limonene, the principal monoterpene in citrus fruit essential oils, is also found in oak, pine, and spearmint. Recently, it has garnered attention as a potential alternative or additive for jet fuel [125,126]. Although limonene is currently produced mainly as a by-product of orange juice manufacturing, the low concentration in natural sources makes its isolation economically unfeasible. Willrodt et al. [127] constructed an *E. coli* strain carrying a dual-plasmid system and performed a two-phase fed-batch operation in a bioreactor. The addition of an inert organic solvent prevented product inhibition, toxic effects, and limonene evaporation losses [90]. Diisononyl phthalate (DINP) has a good partition coefficient and has no detectable effect on *E. coli* growth [128]. When using DINP as the organic phase, the final limonene concentration reached 3630 mg/L [90]. The above examples showed that the appropriate use of biphasic fermentation technology and selection of suitable organic solvents as the SP can effectively enhance the monoterpenes’ productivity.

In the TPF systems, beyond utilizing organic solvents like n-dodecane as the SP, the resin can also be employed as an adsorbent in this phase to enhance monoterpene production. For example, by inducing cells with IPTG and arabinose to increase P450 expression levels and using Amberlite resin to extract the product, the production of perillyl alcohol can be boosted to 105 mg/L [102].

#### 4.1.2. Sesquiterpenes

Sesquiterpenes, with their 15-carbon backbone derived from three isoprene units, exhibit a remarkable diversity in chemical structures and shapes, making them a significant class of terpene known for various structures and functions [129]. These compounds, found in numerous plants, contribute to the unique scents and flavors of many essential oils and have notable biological activities, including the antimalaria drug artemisinin. Unfortunately, the yield of artemisinin extracted from plant extracts is low, increasing the cost of treatments, and is affected by weather and environmental factors. Keasling’s team [130] focused on engineering microbes for the biosynthesis of artemisinin. By reconstructing the MVA pathway from *S. cerevisiae* into *E. coli* and regulating the relevant genes, they successfully produced amorphadiene in *E. coli*, which is the precursor of artemisinin. Using TPF technology, they innovatively used n-dodecane [79], isopropyl myristate [25], and methyl oleate [25] as the organic phase, providing new ideas and methods for the microbial metabolic synthesis of terpenoid compounds. The following Table 2 is a summary of the fermentation results for sesquiterpenes, categorized by various chassis cells, fermentation types and scales, the second phase, and production outputs. This summary specifically highlights the results with the highest yield under each condition; the fermentation data of most sesquiterpenes are attached in Supplementary Materials Table S2.

The isomers α-farnesene and β-farnesene play a crucial role in plant–insect interactions and possess significant economic value in pharmaceuticals, cosmetics, seasonings, and bioenergy [131]. Recent studies have successfully leveraged microbial metabolic engineering for the heterologous production of farnesene. *E. coli*, *S. cerevisiae*, and *Y. Lipolytica* have been successfully engineered for farnesene production [132,133,134,135,136]. Produced through batch fermentation with n-dodecane as the SP in a bioreactor, the production of α-farnesene reached 10.4 g/L [137]. An optimized S. cerevisiae strain in a 20,000 L bioreactor, with polymers and olefins as extractants, significantly increased α-farnesene yield to 130 g/L [138]. You et al. [136] engineered an *E. coli* strain overexpressing β-farnesene with IDI and FPPs, minimizing IPP accumulation. Using n-decane as the organic phase, this strain achieved a final titer of 8.74 g/L. In *Y. lipolytica*, the fusion expression of farnesene synthase and FPPs enhanced α-farnesene synthesis, reduced intracellular accumulation of mevalonate, and yielded 25.55 g/L of α-farnesene in TPF with n-dodecane [139]. Although the yield of α-farnesene through flask fermentation, employing resin as an adsorbent and *Anabaena* sp. as the cell factory, is presently limited to 305.4 μg/L [140], this observation suggests the viability of resin as an SP within the TPF system for sesquiterpene production. This research introduces a new concept and methodology into the realm of fermentation processes for sesquiterpenoid synthesis.

**Table 2:** Summary of the selected highest yield of sesquiterpenes, categorized based on various chassis cells, fermentation scales, the second phase, and production outputs.

Sesquiterpenes	Chassis Cells	Fermentation Types and Scales	Second Phases	Titers (mg/L)	References
amorphadiene	*E. coli*	flask	none	112.2	[130]
		14 mL tube	n-dodecane	300	[141]
		250 mL flask	n-dodecane	1400	[142]
		250 mL bioreactor	n-dodecane	30,000	[16]
	*S. cerevisiae*	250 mL flask	n-dodecane	497	[143]
		2 L bioreactor	n-dodecane	41,000	[79]
		flask	isopropyl myristate	4000	[25]
		2 L bioreactor	methyl oleate	40,000	[25]
	*Y. lipolytica*	250 mL flask	n-dodecane	171.5	[144]
	*R. toruloides*	2 L bioreactor	n-dodecane	36	[145]
	*B. subtilis*	flask	n-dodecane	20	[146]
	*S. elongatus*	100 mL flask	n-hexadecane	19.8	[147]
α-farnesene	*E. coli*	500 mL flask	n-decane	1100	[133]
	*S. cerevisiae*	250 mL flask	n-dodecane	1477.2	[137]
		5 L bioreactor	n-dodecane	10,400	[137]
	*Y. lipolytica*	300 mL flask	n-dodecane	1700	[139]
		1 L bioreactor	n-dodecane	25,550	[139]
	*S. elongatus*	flask	n-dodecane	4.6	[148]
	*Anabaena* sp.	250 mL flask	supelpak 2sv resin columns	0.3054	[140]
	*P. pastoris*	flask	n-dodecane	2560	[149]
β-farnesene	*E. coli*	5 L bioreactor	n-decane	10,310	[150]
		0.5 L flask	n-decane	5290	[151]
	*Y. lipolytica*	2.5 mL tubes	n-dodecane	955	[152]
		2 L bioreactor	n-decane	22,800	[153]
bisabolene	*E. coli*	flask	n-dodecane	1150	[87]
		5 L bioreactor	n-dodecane	9100	[154]
	*S. cerevisiae*	125 mL flask	n-dodecane	994	[155]
		2 L bioreactor	n-dodecane	5200	[79]
	*Synechococcus* sp.	250 mL flask	n-dodecane	0.6	[156]
		3 L bioreactor	n-dodecane	22.5	[157]
	*R. toruloides*	2 L bioreactor	n-dodecane	680	[145]
nerolidol	*E. coli*	5 L bioreactor	n-dodecane	16,000	[32]
	*S. cerevisiae*	250 mL flask	n-dodecane	497	[158]
		5 L bioreactor	n-dodecane	7010	[32]
α-humulene	*E. coli*	2 L bioreactor	Amberlite XAD4 resin	60.2	[24]
		bioreactor	n-dodecane	0.958	[159]
	*S. cerevisiae*	5 L bioreactor	n-dodecane	1726.78	[160]
patchoulol	*E. coli*	5 L bioreactor	n-dodecane	970	[161]
	*S. cerevisiae*	1.1 L flask	n-dodecane	42.1	[162]
		5 L bioreactor	n-dodecane	1632	[163]
valencene	*S. cerevisiae*	300 mL flask	n-dodecane	31	[164]
		3 L bioreactor	n-dodecane	264.6	[165]
	*Y. lipolytica*	flask	n-dodecane	22.8	[166]
	*C. glutamicum*	100 mL flask	n-dodecane	2.41	[167]
	*R. sphaeroides*	250 mL flask	n-dodecane	352	[168]
	*Synechocystis* sp.	flask	isopropyl myristate	9.6	[169]
germacrene A	*E. coli*	flask	none	6.325	[170]
		250 mL flask	n-dodecane	364.26	[171]
		4 L bioreactor	n-dodecane	3520	[64]
	*S. cerevisiae*	flask	n-dodecane	375	[172]
	*Y. lipolytica*	5 L bioreactor	isopropyl myristate	39,000	[78]
	*P. pastoris*	1 L bioreactor	n-dodecane	1900	[173]
	*O. polymorpha*	250 mL bioreactor	n-dodecane	4700	[174]
α-santalene	*E. coli*	1.3 L bioreactor	isopropyl myristate	2916	[175]
	*S. cerevisiae*	2.5 L flask	n-dodecane	92	[176]
		5 L bioreactor	n-dodecane	163	[177]
	*Y. lipolytica*	5 L bioreactor	n-dodecane	27.92	[178]
β-caryophyllene	*E. coli*	25 mL flask	none	100	[179]
		5 L bioreactor	none	1520	[180]
		5 L bioreactor	n-dodecane	5142	[181]
	*S. cerevisiae*	1.3 L bioreactor	n-dodecane	2949.1	[182]
α-cuprenene	*X. dendrorhous*	100 mL flask	n-dodecane	80	[183]
viridiflorol	*E. coli*	250 mL bioreactor	n-dodecane	25,700	[16]
longifolene	*E. coli*	5 L bioreactor	n-decane	382	[184]
(+)-zizaene	*E. coli*	2 L bioreactor	diaion HP20 resin	211	[185]
valerenadiene	*E. coli*	flask	n-decane	62	[186]
protoilludene	*E. coli*	flask	n-decane	1199	[187]
farnesol	*S. cerevisiae*	flask	none	70	[188]
	*E. coli*	flask	methyl oleate	1419	[189]
epi-isozizaene	*E. coli*	4 L bioreactor	n-decane	727.9	[190]
α-isocomene	*E. coli*	bioreactor	n-decane	77.5	[190]
pentalenene	*E. coli*	2.5 L bioreactor	n-decane	780.3	[190]
α-neoclovene	*S. cerevisiae*	1.3 L bioreactor	n-dodecane	487.1	[182]
valerenic acid	*S. cerevisiae*	flask	n-dodecane	4	[191]
zerumbone	*S. cerevisiae*	5 L bioreactor	n-dodecane	40	[192]
prespatane	*R. toruloides*	2 L bioreactor	n-dodecane	1173.6	[77]
santalols	*S. cerevisiae*	5 L bioreactor	n-dodecane	1300	[193]
z-α-Santalol	*S. cerevisiae*	5 L bioreactor	n-dodecane	1200	[193]
zerumbone	*S. cerevisiae*	5 L bioreactor	n-dodecane	40	[192]

β-elemene is a sesquiterpene extracted from *Curcuma aromatica* Salisb. ‘*Wenyujin*’ and is one of the most widely used antitumor drugs for the treatment of various cancer tumors in China [194]. The heterologous MVA pathway and cyanobacterial enzyme genes were concurrently introduced into *E. coli*, resulting in a β-elemene yield of 6325.5 µg/L in shaking bottles [170]. However, this yield is insufficient for industrial production. Recent studies have identified efficient synthases from algae and integrated key pathway enzymes, export genes, and translational engineering to implement TPF technology in bioreactors with n-dodecane as the SP, achieving a β-elemene yield of 3.52 g/L [64]. *Y. lipolytica*, serving as an exceptional cell factory, has been engineered to reconstruct the endogenous mevalonate pathway and regulate lipid metabolism, resulting in a β-elemene titer of 39 g/L in a bioreactor containing an isopropyl myristate organic phase [78].

#### 4.1.3. Diterpenes

Diterpenes, with 20 carbon atoms from four isoprene units, exhibit vast structural diversity and a wide range of biological activities, making them crucial in pharmaceuticals, food additives, fragrance synthesis, and agriculture [195]. Paclitaxel (Taxol^®^), a compound found in the bark of the Pacific yew tree, stands out for its effectiveness against breast and ovarian cancers. The overexpression of enzymes in the MEP pathway and paclitaxel synthase in *E. coli* led to the successful creation of a strain capable of producing taxadiene, a key precursor of paclitaxel, with a yield of 1 g/L achieved through two-phase fed-batch fermentation using n-dodecane as the SP [196]. In the case of *S. cerevisiae*, both liquid–liquid TPF with n-dodecane and solid–liquid fermentation using silica gel as an adsorbent were effective in enhancing taxadiene yield, which gave the yield of 129 mg/L [197] and 8 mg/L [66], respectively. These findings underscore the efficacy of TPF technology in enhancing productivity (Table 3).

Another example of fermentation paclitaxel has been discussed above, where the mutant strains of *A. fumigatus* and *A. tenuissima* were immobilized by five different entrapment materials and successfully applied for production enhancement of paclitaxel. The paclitaxel titers obtained by the immobilized mycelia of the respective mutants, 694.67 and 388.65 μg/L, were promising for fungal production of paclitaxel [52]. Thus, the immobilized cell technology has shown considerable potential for application in the industrial-scale production of paclitaxel through biotechnological processes.

In the production of miltiradiene using *S. cerevisiae* as the cell factory, the use of n-dodecane as the SP increased the yield by up to ten times, archiving at 3.5 g/L [198]. By strengthening upstream pathways, regulating central carbon metabolism and cofactor supply, fusing and truncating terpenoid synthase genes, knocking out related regulatory factors, and using TPF technology with n-hexane, the yield of sclareol in the bioreactor reached 11.4 g/L [199].

**Table 3:** Summary of the selected highest yield of diterpenes, categorized based on various chassis cells, fermentation scales, the second phase, and production outputs.

Diterpenes	Chassis Cells	Fermentation Types and Scales	Second Phases	Titers (mg/L)	References
miltiradiene	*S. cerevisiae*	5 L bioreactor	none	488	[195]
		10 mL flask	n-dodecane	550	[198]
		5 L bioreactor	n-dodecane	3500	[198]
taxadiene	*E. coli*	flask	none	1.3	[200]
		2 L flask	n-dodecane	570	[201]
		1 L bioreactor	n-dodecane	1020	[196]
	*S. cerevisiae*	500 mL bioreactor	none	33	[202]
		500 mL flask	RP18 silica gel	8	[66]
		500 mL bioreactor	n-dodecane	129	[197]
	*A. fumigatus*	250 mL flask	immobilization	0.694	[52]
	*A.tenuissima*	250 mL flask	immobilization	0.388	[52]
oxygenated taxane	*S. cerevisiae*	1 L bioreactor	n-dodecane	78	[203]
ent-Kaurene	*E. coli*	1 L bioreactor	none	578	[204]
		3 L bioreactor	n-dodecane	624	[205]
	*R. toruloides*	2 L bioreactor	n-dodecane	1400	[206]
geranylgeraniol	*S. cerevisiae*	bioreactor	none	3300	[207]
		flask	n-dodecane	772.98	[208]
		5 L bioreactor	n-dodecane	5070	[208]
steviol	*E. coli*	2q L bioreactor	none	1100	[209]
		3 L bioreactor	n-dodecane	38.4	[205]
sclareol	*E. coli*	bioreactor	n-dodecane	1500	[210]
	*S. cerevisiae*	100 mL flask	n-dodecane	750	[211]
		0.4 L bioreactor	n-hexane	11,400	[199]
levopimaradiene	*E. coli*	3 L bioreactor	n-dodecane	700	[212]
levopimaric acid	*S. cerevisiae*	5 L bioreactor	n-dodecane	400.3	[213]
retinoids	*E. coli*	14 mL tube	n-dodecane	33	[214]
retinol	*S. cerevisiae*	5 L bioreactor	n-dodecane	2349	[215]
	*Y. lipolytica*	5 L bioreactor	n-dodecane	4860	[216]
cis-abienol	*E. coli*	bioreactor	isopropyl myristate	634	[217]
13R-manoyl oxide	*S. cerevisiae*	5 L bioreactor	n-dodecane	3000	[218]
forskolin	*S. cerevisiae*	5 L flask	n-hexane	40	[219]
gibberellic acid 3	*Y. lipolytica*	24-roundwell plates	none	12.8	[220]
gibberellic acid 4	*Y. lipolytica*	24-roundwell plates	none	17.3	[220]
carnosic acid	*S. cerevisiae*	30 mL flask	none	25	[221]
		5 L bioreactor	none	75.2	[221]
rubusoside	*S. cerevisiae*	250 mL bioreactor	none	1400	[222]
rebaudiosides	*S. cerevisiae*	250 mL bioreactor	none	132.7	[222]

#### 4.1.4. Triterpenes and Tetraterpenes

Triterpenes and tetraterpenes, composed of six and eight isoprene units, respectively, play diverse roles in nature and human applications [223,224,225]. Triterpenes are recognized for their biological activities, often utilized in traditional medicine for their anti-inflammatory, antiviral, and anticancer properties. Tetraterpenes are best known for their presence in colorful plant pigments, such as carotenoids [225]. Most economically valuable triterpenoids are water-soluble, featuring hydrophilic groups like carboxyl or sugar moieties, enabling their dissolution in the aqueous phase during microbial fermentation such as ginsenosides, thus bypassing the need for an extraction solvent phase. This eliminates the need for an extract SP (Table 4). Conversely, lipophilic triterpenoids, such as squalene—used in cosmetics, dietary supplements, and as a vaccine adjuvant—require TPF for biosynthesis. Employing yeast with n-dodecane as the solvent phase has yielded significant squalene production (207.02 mg/L) [137]. Similarly, protopanaxadiol, the precursor to ginsenosides, has been biosynthesized using yeast as a cell factory, with methyl oleate or n-dodecane serving as in situ extraction solvents, resulting in a yield of 1189 mg/L [226]. In contrast, tetraterpenoids, particularly plant pigments, are fat-soluble substances due to their hydrophobic structures. β-Carotene, a naturally occurring red-orange pigment and one of the important tetraterpenoids, plays a crucial role in maintaining vision, skin health, and a properly functioning immune system owing to its conversion into vitamin A. In general, β-carotenoids produced by microorganisms are intracellularly stored, not released outside the cell. For instance, *Y. lipolytica* has a large intracellular organelle for lipid storage, referred to as the lipid body. A literature investigation reveals that only lycopene has been subject to TPF in the cells of *Y. lipolytica*, using n-dodecane as the organic solvent, with a yield of 4.2 g/L [227] (Table 4). However, the purpose of adding n-dodecane was solely to minimize the evaporation of isoprenol, which is an additional substrate, and not for in situ extraction.

## 5. Factors Influencing TPF Systems

Factors influencing the efficiency and effectiveness of TPF systems are varied and complex, impacting the overall success of the fermentation process. When selecting materials for the SP, factors such as chemical stability, potential toxicity, interactions with aqueous phase components, solubility, and the ability to stabilize released products must be considered. The ideal SP choice varies depending on the specific product being accumulated.

### 5.1. Solvent or Adsorbent as the Second Phase

In TPF systems, organic solvents or macroporous resins are typically employed as the SP, especially in the fermentation of terpenes. ILs, while available, are less suitable due to their significant toxicity towards microbial cells [271]. The impact of physical parameters during fermentation, such as high-speed stirring, and post-fermentation processing issues also needs to be evaluated to ensure the SP does not disrupt cell integrity or impede industrial scalability. The selection of solvents or adsorbents as the SP greatly influences the efficiency of the system. Ensuring compatibility with the microbial culture and the aqueous phase is crucial. Similarly, the choice of microbial strain for fermentation is vital, as different strains possess distinct tolerances and metabolic capabilities, which significantly impact the yield and efficiency of the fermentation process.

#### 5.1.1. Solvent Selection Considerations

Solvent toxicity is a primary concern when selecting an SP. The chosen solvent should be minimally toxic to microbial cells, and its biocompatibility and biodegradability are important considerations. Biodegradability means that the microbial cells do not degrade the organic solvent during the fermentation. For example, the solvents may potentially be degraded and used as a carbon source by some microorganisms [272,273]. Regarding biocompatibility, it has been widely accepted that the solvent tolerance of microorganisms correlates with the *log p* parameter, where *p* is the 1-octanol/water partition coefficient in the two-phase system [29,274,275,276]. In general, solvents with *log p* values below 2 are in general toxic, and organic solvents with a *log p* > 4 have been found to be compatible with microbial cells [275]. However, the tolerance of a particular strain to an organic solvent is not always evaluated in a straightforward way and can be influenced by medium composition, cultivation conditions, and inoculum history. For instance, Philipp Demiling et al. [277] screened 18 different kinds of organic solvents for biocompatibility and biodegradability and found that ethyl decanoate showed high biocompatibility and negligible biodegradability for the biosynthesis of rhamnolipids by *Pseudomonas putida* KT2440. Selecting an appropriate solvent is pivotal for TPF efficiency and yield, necessitating a comprehensive evaluation of potential solvents based on microbial strain characteristics and fermentation conditions. In Jacek Kujawski et al.’s [278] article, they present an online tool—the ALOGPS 2.1 program—for the calculation of the *log p* values of compounds.

The target product’s solubility in the solvent, ensuring efficient extraction from the fermentation broth, is another critical factor. For example, the *log p* values of oleic acid and DBP are 7.7 and 5.4, respectively, and the partition coefficients of paclitaxel in these solvents are 154 and 236, respectively [21]. In addition, the solvent should have low volatility; solvents with low volatility are preferred to minimize losses due to evaporation and to reduce the risk of flammability and other safety hazards.

As indicated in Table 1, Table 2, Table 3 and Table 4 and Supplementary Materials, the majority of terpene fermentations utilize organic solvents as the SP, with n-dodecane being the most commonly used. Consequently, Table 5 was compiled, summarizing the nine different types of organic solvents selected for terpene compounds, including crucial *log p* values, as well as boiling points, which are essential for further discussion. However, we found that the *log p* results in different values, whether experimental or predicted, for the same chemical in different literatures. Despite these disparities, the specific numerical values will not differ significantly.

#### 5.1.2. Adsorbent Selection Considerations

Compared to organic solvents, adsorbents offer lower toxicity and fewer biocompatibility issues. However, several additional factors must be considered, including the adsorption of components from the culture medium, the diversity of resin types, and their physical properties.

Adsorption of components in culture medium: The potential for adsorbents to absorb nutrients or other components from the culture media is a critical concern. For instance, aromatic acids may bind specifically to polystyrenic adsorbents. A notable example is the XAD-16 resin, known to bind methyl oleate, a primary carbon source in *Myxococcus xanthus* for epothilone production [285].

Diversity of resins: Adsorbents considered for in situ adsorption in fermentation may vary in chemical and physical properties, such as polymer chemistry, surface area, particle size, and pore size, as reviewed by Thomas Phillips et al. [22]. Thus, the product’s polarity and the resin properties are essential considerations. Resins can be categorized based on their adsorption mechanism—physical adsorption, which occurs without altering the chemical properties of the adsorbate, and chemical adsorption, involving chemical bond formation between the adsorbate and adsorbent [286]. Additionally, polymeric adsorbents are classified by their composition and functionality, including nonionic, anionic, cationic, and affinity resins.

In recent years, ion exchange resins have also been used in in situ fermentation [287]. Comprising tiny, porous beads made from an organic polymer matrix, these resins are functionalized with active groups to selectively bind and exchange specific ions in the fermentation system. The ion exchange process facilitates separation based on ion concentration and resin characteristics, with applications ranging from organic acid to amino acid separation. Examples of the latter are the anions of organic acids, produced by fermentation [288], e.g., lactic acid, citric acid, some amino acids, etc. A few reviews of the ion exchange resins have been published [287,289]. Thus, in this part, we only illustrated one typical example of lactic acid production by the application of ion exchange resins. Lactic acid is a monomer in the production of biodegradable polylactic acid (PLA), which is a well-known sustainable bioplastic material. Ahasa Yousuf and colleagues [290] employed Amberlite IRA-67, a weak ion exchange resin, for in situ extraction of lactic acid from 7-day dark fermentation broths of food waste. IRA-67 showed a maximum acid removal of 74%. However, the application in terpenoid fermentation is limited by the pH tolerance of terpenoids and the microbial resistance to acid or alkali.

Physical properties of resins: The high-speed agitation involved in fermentation processes poses a risk to the integrity of resin beads. While resin particle integrity may not directly impact adsorption capability, the initial recovery step often involves sieving, where physical degradation can lead to separation losses [22]. Frykman and his colleagues [285] compared two possible modes of resin bead breakage: collisions between resin particles and collisions between resin particles and the agitator. Theoretically, at an agitation speed of 800 rpm, these models suggest that most resin particle breakage is more likely caused by impeller blades hitting the beads rather than collisions between the beads themselves. The relationship between agitation rate and bead breakage was then experimentally evaluated using laser diffraction particle sizing to measure the size distribution of XAD-16 resin particles. Bead breakage was found to be negligible in the first 3 days when the agitation rate was 600 rpm (impeller tip speed = 2.0 m/s). However, after 10 days of agitation at 800 rpm (impeller tip speed = 2.7 m/s), the particle size was bimodally distributed, with 29% of particles having a diameter less than 250 μm and the remaining particles relatively unchanged (mean diameter of 700 μm) [22,285]. Thus, resin particle breakage (like cell breakage) may be influenced by other factors such as rheology and the length of fermentation. Particularly in industrial scale-up processes, this phenomenon should be given more attention. Although many considerations when selecting an appropriate adsorption resin have been discussed above, the statistical data in Table 1, Table 2, Table 3 and Table 4 showed that resin usage in terpene fermentation is exceptionally limited, pointing to a gap in the targeted research. Thus, selecting an appropriate resin for terpene fermentation in the TPF process relies on the existing literature and optimization through screening experiments.

### 5.2. Concentration and Timing of Second Phase Addition

Due to the different mechanisms of the effects of different concentrations of adsorbents and organic solvents on microbial TPF, a summary and overview of the addition concentrations of adsorbents and organic solvents are discussed separately below.

Concentration of adsorbent: Determining the optimal concentration of the adsorbent is crucial in TPF and requires experimental optimization. Typically quantified by weight per volume, resin concentrations in the literature range from 0.5% to 20% [22]. Insufficient adsorbent may lead to inadequate product sequestration, while excessive amounts can negatively impact cell growth and product concentration. For example, in the production of 10′-deoxymethynolide, the highest yield, reaching 280 mg/L, was obtained by adding HP2MGL resin at a concentration of 10% (ranging from 4% to 20%) [291]. Excessive resin not only adsorbs fermentation products from the fermentation liquid but also interrupts the adsorption of nutrients by producing microbes in the fermentation system, thereby inhibiting microbial growth and reducing the yield of the target compound.

In the TPF of PDTs, only a few volatile monoterpenes or sesquiterpenes selected resins as the adsorbents, which is summarized in Table 1, Table 2, Table 3 and Table 4. The concentration range of the resins is from 4% to 10%. Semra Alemdar et al. [24] employed hydrophobic resin Amberlite R XAD4 (10%) as the SP, increasing α-humulene yield by 2310% to 60.2 mg/L, using *E. coli* as the cell factory in a 2 L bioreactor. In the study of biosynthesis taxadiene by *S. cerevisiae*, Benedikt Engels et al. [66] chose 0.5% *w*/*v* RPC18 silica gel for product adsorption, resulting in a 40-fold increase in taxadiene to 8.7 mg/L. However, few studies investigate the effect of the concentration of resin addition on the TPF of PDTs.

Concentration of organic solvents: Organic solvents primarily act on the plasmalemma, affecting solute transport, energy maintenance, and intracellular homeostasis. The critical concentration of organic solvents is approximately 200 mM, with toxicity related to membrane distribution rather than specific chemical structure [292]. Concentrations that cause similar toxic effects are alike for compounds displaying different *log p* values, as can be seen from the evaluation of the biocompatible properties of different solvents discussed above. Hence, the membrane concentration of the solvent depends on key factors including the solvent’s concentration in the water phase, its partitioning from water into the membrane, and the volume ratio of the two liquid phases [293]. Therefore, the solvent’s membrane concentration can be calculated when the water phase concentration is known. Conventionally, organic solvents range from 4% to 20% in TPF based on prior studies.

Timing of second phase addition: Adding adsorbents or organic solvents as the SP to the fermentation system for in situ extraction of the product requires careful consideration of the timing. The efficiency of the process is influenced by when the SP is introduced. The considerations of the addition time for these two second terms will be discussed together.

The time for adding the SP needs to consider the growth cycle of microbial cells [294]. The SP should ideally be added at a point where the cells are most active and productive. This is often during the exponential growth phase [294]. When utilizing organic solvents as SPs in the experiments, they are typically introduced during the stage of inducing specific proteins’ expression in cells. Taking *E. coli* as an example, when the cell growth reaches a certain optical density (OD) range, such as between OD values of 1 and 2, the organic solvents are added simultaneously with inducers like isopropyl β-d-thiogalactoside (IPTG) [295]. This is because the introduction of the inducer activates specific proteins within the cells, initiating the biosynthesis of the product [296]. Similarly, in the *S. cerevisiae* system, organic solvents are introduced along with inducers, such as galactose, required for the *GAL* promoters [297], past a certain level of cell proliferation. In some cases, if the constitutive promoters are employed in *S. cerevisiae*, the addition of organic solvents is timed to coincide with a particular OD value when product biosynthesis commences.

The above discussion about addition time of the SP serves as a basic guideline. The essential principle is to delay the addition until the microorganisms attain a certain biomass. The timing for the SP addition is not static but is established through meticulous optimization, considering the microbial factory’s metabolic state and other pertinent factors. Taking the current highest yield production of β-elemene as an example, Qi Liu et al. [78] chose to add 10% isopropyl myristate (200 mL in a 2 L medium) after culturing *Y. lipolytica* for 24 h and then supplemented another 10% of IPM when the fermentation progressed to 96 h. This flexible adjustment of the addition strategy helps to improve the production efficiency of β-elemene. As for the use of adsorbents, they are typically added to the bioreactor along with the culture medium during the sterilization process due to their significantly lower cellular toxicity compared to organic solvents, facilitating earlier addition without impeding cell growth.

This timing is crucial to minimize interference with cell growth while ensuring their effective role in product synthesis onset. As discussed above, the added phase can alleviate feedback inhibition. If the product inhibits microbial activity, adding the SP early to remove the product from the system can be beneficial. Therefore, the optimal timing for the addition of the SP in a TPF system depends on a complex interplay of biological, chemical, and operational factors.

### 5.3. Economic Considerations and Downstream Processing

The cost of the solvent or adsorbent used in the SP is a significant factor for further industrial application. As discussed above, n-dodecane is a commonly used organic solvent but is not an economically viable extracting solvent. In the research by Gui Hwan Han et al. [154], natural vegetable oils, like canola, olive, corn, and soybean oil, showed a similar in situ extraction effect as n-dodecane, with extraction yields of (−)-α-bisabolol ranging from 96.6 to 98.8%, comparable to that of n-dodecane (99.5%). These findings suggest that vegetable oils can serve as natural, cost-effective, and biodegradable extractors during fermentation, reducing production costs. Considering the discussion on organic solvents above, it is feasible to explore more cost-effective alternatives. However, some microorganisms may use natural vegetable oil as a carbon source [298,299], potentially impacting in situ extraction. Therefore, it is essential to consider carbon source supplementation, especially in fed-batch fermentation, where constant monitoring of carbon source consumption is necessary to supplement new sources before depletion.

Downstream processing economic considerations include the costs associated with product recovery and purification, as well as the recovery, regeneration, and recycling associated with the SP. In fermentation processes involving organic solvents, the SP entails multiple critical procedures such as centrifugation for organic solvent collection, solvent concentration, and product purification like distillation. Although organic solvents are commonly used in microbial fermentation of PDTs (Table 1, Table 2, Table 3 and Table 4), they often form emulsion-like mixtures with water [300], especially under vigorous stirring in fermenters. While emulsification enhances substance transfer and biological reaction efficiency, it can pose operational challenges and increase processing costs by reducing recovery efficiency.

Many different downstream technologies have been applied to the separation and purification of terpenes, such as chromatography, distillation, and ion exchange. For instance, preparative HPLC and high-speed countercurrent chromatography are highly adept at separating terpenoids exhibiting high polarity, such as the valuable ginsenosides [301]. These methods are preferred as they minimize sample loss resulting from irreversible adsorption.

Distillation equipment, such as a distillation column, is suitable for volatile components like monoterpenes and sesquiterpenes. By consulting Table 5 for the boiling points of common organic solvents and details on high-value volatile terpenes, an optimal solvent can be chosen. For example, β-elemene is extracted from the plant *C. aromatica* and purified by the distillation column in the industry. The boiling point of β-elemene is predicted as 252.1 ± 35.0 °C at 760 mmHg using ACD/Labs Percepta Platform-PhysChem Module in ChemSpider. If the actual boiling point value of β-elemene is the lowest value of this predicted data, which is 217.1 °C, then using n-dodecane or methyl oleate as the SP for the TPF is not appropriate. The boiling point of the β-elemene is too close to the organic solvent, which will make more steps in the purification of distillation. Although the boiling point data of most volatile terpenes are based on model predictions, in Dustin Barton and James Chick’s paper [302], they have measured the enthalpy of evaporation of a series of sesquiterpenes, which can help to calculate and predict the boiling points of sesquiterpenes based on these data using the Clapeyron equation, and provide assistance for the selection of subsequent organic solvents. Employing resins as the SP simplifies and enhances the efficiency of subsequent processes. This advantage is primarily due to easy filtration collection and the convenience of dissolution using suitable organic solvents. In the research conducted by Francisco Aguilar et al. [185], they tested seven different solvents for elution of (+)-zizaene from the adsorber Diaion HP2; among them, isooctane showed the best elution effect. Economically, selecting resins as the SP offers advantages, with higher recovery and reuse rates compared to organic solvents, which typically incur losses post-processing.

## 6. Conclusions and Future Perspectives

In summary, TPF systems present a promising approach for microbial production of PDTs, providing advantages such as reducing microbial cell toxicity, facilitating product recovery, and simplifying post-fermentation processes. Despite these benefits, challenges like enhancing yields, improving recovery rates, and managing costs persist, highlighting areas for further development.

Current research extensively covers the use of organic solvents in TPF, but there is a lack of depth in selecting and optimizing solvents for specific terpenes. Similarly, the potential economic and processing benefits of resins remain underexplored, indicating a significant opportunity for future studies to focus on resin-based systems.

In the industrial production of terpenes, downstream-processing methods are still insufficient. For instance, the treatment of emulsification phenomena and the question of whether only distillation methods are suitable for the post-processing of volatile terpenes have not been fully explored in the published related fermentation research. This is particularly evident in Linhao Chen’s review [301], where only about 34 types of terpenes are discussed. Compared to the many terpenes listed in Table 1, Table 2, Table 3 and Table 4, it is clear that more work is needed to deepen the understanding of this field.

Emerging triphasic and multiphasic fermentation methods, such as the notable study achieving a yield of 64.6 g/L in butanol fermentation by a triphasic fermentation system consisting of immobilized cells, medium, and extraction agent, underscore the potential for innovative approaches in this field [53]. By addressing these highlighted gaps and leveraging new technologies, we can advance terpene fermentation toward higher efficiency and broader application. This overview aims to spur further innovation and research in TPF systems, emphasizing the need for a concerted effort to overcome existing hurdles and explore new fermentation strategies, ultimately driving the field toward more efficient and sustainable practices.

## Figures and Tables

**Table 1:** Summary of the fermentation results for monoterpenes, categorized by various chassis cells, fermentation scales, the second phase, and production outputs. This summary specifically highlights the results with the highest yield under each condition.

Monoterpenes	Chassis Cells	Fermentation Types and Scales	Second Phases	Titers (mg/L)	References
geraniol	*E. coli*	5 L bioreactor	none	78.8	[65]
		flask	n-decane	1119	[81]
		flask	isopropyl myristate	2102.5	[83]
		10 L bioNreactor	isopropyl myristate	13,190	[80]
	*S. cerevisiae*	flask	none	36.04	[84]
		5 L bioreactor	isopropyl myristate	1680	[85]
		1 L bioreactor	n-dodecane	1690	[82]
	*C. glutamicum*	250 mL flask	n-dodecane	15.2	[86]
limonene	*E. coli*	flask	n-dodecane	605	[87]
		250 mL flask	isopropyl myristate	1290	[88]
		250 mL flask	diisononyl phthalate	37.8	[89]
		3.1 L bioreactor	diisononyl phthalate	3630	[90]
	*S. cerevisiae*	flask	none	62.31	[91]
		flask	isopropyl myristate	2230	[92]
		flask	n-dodecane	2580	[57]
		3 L bioreactor	n-dodecane	2630	[93]
	*R. toruloides*	250 mL tube	n-dodecane	393.5	[94]
		250 mL flask	n-dodecane	358.1	[95]
	*Ashbya gossypii*	40 mL flask	n-dodecane	336.4	[96]
	*Y. lipolytica*	flask	n-dodecane	23.56	[97]
		1.5 L bioreactor	n-dodecane	165.3	[98]
	*Synechococcus* sp.	250 mL flask	n-dodecane	6.7	[99]
	*cyanobacteria*	flask	isopropyl myristate	16.4	[100]
perillyl alcohol	*E. coli*	5 L bioreactor	n-dodecane	87	[101]
		flask	anion exchange column with Amberlite resin	105	[102]
linalool	*E. coli*	500 mL flask	none	63	[103]
		flask	n-nonane	1054	[104]
		250 mL flask	n-dodecane	505	[105]
		flask	isopropyl myristate	1250	[106]
		1.3 L bioreactor	isopropyl myristate	1523.2	[107]
	*S. cerevisiae*	500 mL flask	none	0.095	[108]
		2 L bioreactor	none	23.45	[109]
		flask	isopropyl myristate	80.9	[110]
	*Y. lipolytica*	flask	n-dodecane	6.96	[111]
		flask	isopropyl myristate	109.6	[112]
	*Pantoea ananatis*	tube	isopropyl myristate	5600	[113]
		bioreactor	isopropyl myristate	10,900	[114]
cineole	*E. coli*	flask	n-nonane	116.8	[115]
		flask	n-dodecane	653	[105]
	*S. cerevisiae*	bioreactor	none	1100	[116]
sabinene	*E. coli*	5 L flask	none	2650	[117]
		5 L bioreactor	none	150	[118]
	*S. cerevisiae*	flask	n-dodecane	17.5	[119]
pinene	*E. coli*	5 L bioreactor	none	970	[120]
		50 mL flask	n-dodecane	166.5	[121]
	*S. cerevisiae*	50 mL flask	isopropyl myristate	11.7	[122]
	*C. glycerinogenes*	flask	n-dodecane	6	[123]
myrcene	*E. coli*	250 mL flask	n-dodecane	58.19	[124]
		1 L flask	isopropyl myristate	1250	[106]

**Table 4:** Summary of the fermentation results for triterpenes and tetraterpenes, categorized by various chassis cells, fermentation scales, the second phase, and production outputs. This summary specifically highlights the results with the highest yield under each condition.

Triterpenes and Tetraterpenes	Chassis Cells	Fermentation Types and Scales	Second Phases	Titers (mg/L)	References
squalene	*S. cerevisiae*	5 L bioreactor	none	9472	[228]
		5 L bioreactor	n-dodecane	207.02	[137]
ambrein	*E. coli*	flask	none	2.6	[229]
	*P. pastoris*	5 L bioreactor	none	100	[230]
betulin	*S. cerevisiae*	5 L flask	none	59.5	[231]
gypsogenin	*S. cerevisiae*	bioreactor	none	146.84	[232]
lupeol	*S. cerevisiae*	flask	none	200.1	[233]
α-amyrin	*S. cerevisiae*	20 mL flask	none	213.7	[234]
		5 L bioreactor	none	1100	[234]
β-amyrin	*S. cerevisiae*	5 L bioreactor	none	138.8	[235]
		tube	none	6	[236]
ursolic acid	*S. cerevisiae*	10 mL flask	none	101.4	[237]
		bioreactor	none	123.27	[238]
betulinic acid	*S. cerevisiae*	50 mL flask	none	91.6	[237]
		5 L bioreactor	none	1000	[231]
	*Y. lipolytica*	flask	isopropyl myristate	51.87	[239]
morolic acid	*S. cerevisiae*	50 mL flask	none	68.3	[237]
		bioreactor	none	34.1	[237]
oleanolic acid	*S. cerevisiae*	5 L bioreactor	none	606.9	[240]
	*S. cerevisiae*	flask	none	186.1	[240]
ganoderic acid	*S. cerevisiae*	flask	none	14.5	[241]
maslinic acid	*S. cerevisiae*	5 L bioreactor	none	384	[242]
corosolic acid	*S. cerevisiae*	5 L bioreactor	none	141	[242]
alphitolic acid	*S. cerevisiae*	5 L bioreactor	none	23	[242]
quillaic acid	*S. cerevisiae*	bioreactor	none	314.01	[232]
polpunonic acid	*S. cerevisiae*	tube	none	1.4	[243]
glycyrrhetinic acid	*S. cerevisiae*	5 L bioreactor	none	18.9	[244]
dammarenediol-II	*S. cerevisiae*	7 L bioreactor	none	15,000	[245]
		50 mL flask	none	211.52	[246]
		7.5 L bioreactor	n-dodecane /methyl oleate	1548	[226]
	*E. coli*	250 mL flask	none	8.63	[247]
protopanaxadiol	*S. cerevisiae*	250 mL flask	none	17.2	[248]
		10 L bioreactor	none	9054.5	[249]
		7.5 L bioreactor	n-dodecane /methyl oleate	1189	[226]
protopanaxatriol	*S. cerevisiae*	250 mL flask	none	15.9	[248]
ginsenoside Rh2	*S. cerevisiae*	50 mL flask	none	16.9	[250]
		10 L bioreactor	none	2250	[249]
ginsenoside Rg3	*S. cerevisiae*	1.5 L bioreactor	none	1.3	[251]
		50 mL flask	none	51.8	[250]
ginsenoside RF1	*S. cerevisiae*	flask	none	42.1	[252]
ginsenoside Rh1	*S. cerevisiae*	flask	none	92.8	[252]
β-carotene	*E. coli*	flask	none	503	[253]
		5 L bioreactor	none	3200	[254]
	*S. cerevisiae*	2 mL tube	none	477.9	[255]
lycopene	*E. coli*	5 mL tube	none	77.85	[256]
		7 L bioreactor	none	3520	[257]
	*S. cerevisiae*	7 L bioreactor	none	2370	[258]
	*Y. lipolytica*	3 L bioreactor	none	4200	[227]
	*Mucor circinelloides*	500 mL flask	none	54,000	[259]
	*R. rubrum*	100 mL flask	none	15	[260]
	*Rhodobacter sphaeroides*	250 mL flask	none	66.05	[261]
	*Haloferax mediterranei*	5 L flask	none	429.41	[262]
	*P. pastoris*	4 L bioreactor	none	73.9	[263]
		3 L flask	none	714	[264]
astaxanthin	*E. coli*	5 L bioreactor	none	1820	[265]
crocetin	*S. cerevisiae*	5 L bioreactor	none	6.278	[266]
zeaxanthin	*E. coli*	250 mL flask	none	43.46	[267]
		5 L bioreactor	none	722.46	[268]
	*S. cerevisiae*	tube	none	1.5	[269]
	*Pseudomonas putida*	flask	none	51.3	[270]

**Table 5:** Summary of organic solvents in terpene water–organic TPF, detailing the names, CAS numbers, chemical structures, molecular formulas, and critical physical properties, including *log p* values and boiling points.

Name	CAS Number	Chemical Structure and Formula	*Log p*	Boiling Point ^b^ (°C)	References
n-dodecane	112-40-3	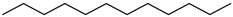 C_12_H_26_	6.6	216.3	[279]
isopropyl myristate	110-27-0	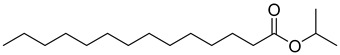 C_17_H_34_O_2_	7.02	315.0	[280]
n-decane	124-18-5	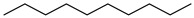 C_10_H_22_	5.6	174.1	[281]
oleyl alcohol	143-28-2	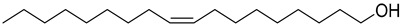 C_18_H_36_O	7.5	305-370	[281]
2,2,4-trimethylpentane	540-84-1	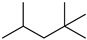 C_8_H_18_	4.49 ^a^	99.2	[278]
n-hexane	110-54-3	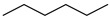 C_6_H_14_	3.5	68.8	[281]
methyl oleate	112-62-9	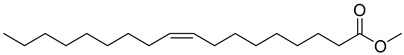 C_19_H_36_O_2_	11.2	218.5	[282]
n-nonane	111-84-2	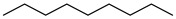 C_9_H_20_	5.65	150.7	[283]
diisononyl phthalate	28553-12-0	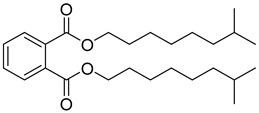 C_26_H_42_O_4_	9.37	77.7	[284]

^a^ The *log p* value is calculated by ALOGPS 2.1, ^b^ the boiling point data for these compounds were retrieved from PubChem. According to PubChem, these boiling points were acquired at 760 mmHg.

## Data Availability

Data sharing is not applicable.

## Supplementary Materials

The following supporting information can be downloaded at: https://www.mdpi.com/article/10.3390/molecules29051127/s1, Table S1: Summary of the fermentation results for monoterpenes, categorized by various chassis cells, fermentation types, the second phase, and production outputs; Table S2: Summary of the fermentation results for sesquiterpenes, categorized by various chassis cells, fermentation types, the second phase, and production outputs; Table S3: Summary of the fermentation results for diterpenes, categorized by various chassis cells, fermentation types, the second phase, and production outputs; Table S4: Summary of the fermentation results for triterpenes, categorized by various chassis cells, fermentation types, the second phase, and production outputs; Table S5: Summary of the fermentation results for tetraterpenes, categorized by various chassis cells, fermentation types, the second phase, and production outputs.
