# Supplementary material for: Two-Phase Fermentation Systems for Microbial Production of Plant-Derived Terpenes
Source: Molecules. 2024 Mar 2;29(5):1127. doi: 10.3390/molecules29051127 (PMC10934027; doi:10.3390/molecules29051127)
Supplement: Supplementary file 1 [file molecules-29-01127-s001.zip › molecules-2893530-supplementary.pdf]

Two-Phase Fermentation Systems for Microbial Production of Plant-Derived Terpenes

Table S1. Summary of the fermentation results for monoterpenes, categorized by various chassis cells, fermentation types, the second phase, and production outputs.

Monoterpenes	Chassis cells	Fermentation types	Second phases	Titers (mg/L)	References
geraniol	*E. coli*	bioreactor	none	78.8	[1]
		flask	isopropyl myristate	2102.5	[2]
		bioreactor	isopropyl myristate	13190	[3]
		bioreactor	isopropyl myristate	2000	[1]
		flask	n-decane	182.5	[4]
		flask	n-decane	1119	[5]
		flask	n-decane	300	[6]
	S. cerevisiae	flask	none	5	[7]
		flask	none	36.04	[8]
		bioreactor	n-dodecane	227	[9]
		bioreactor	n-dodecane	293	[10]
		bioreactor	n-dodecane	1690	[11]
		bioreactor	isopropyl myristate	1680	[12]
	C. glutamicum	flask	n-dodecane	15.2	[13]
limonene	*E. coli*	flask	n-dodecane	435	[14]
		flask	n-dodecane	605	[15]
		flask	n-dodecane	214	[16]
		flask	isopropyl myristate	1290	[17]
		flask	diisononyl phthalate	37.8	[18]
		bioreactor	diisononyl phthalate	2700	[19]
		bioreactor	diisononyl phthalate	3630	[20]
	S. cerevisiae	flask	none	62.31	[21]
		flask	n-dodecane	0.12	[22]
		flask	n-dodecane	1.48	[23]
		flask	n-dodecane	2580	[24]
		flask	n-dodecane	166	[25]
		flask	n-dodecane	76	[26]
		bioreactor	n-dodecane	2630	[27]
		flask	isopropyl myristate	917	[28]
		flask	isopropyl myristate	2230	[29]
	R. toruloides	tube	n-dodecane	393.5	[30]
		flask	n-dodecane	358.1	[31]
	A. gossypii	flask	n-dodecane	336.4	[32]

	Y. lipolytica	flask	n-dodecane	23.56	[33]
		bioreactor	n-dodecane	165.3	[34]
	Synechococcus sp.	flask	n-dodecane	4	[35]
		flask	n-dodecane	6.7	[36]
	cyanobacteria	flask	isopropyl myristate	16.4	[37]
perillyl alcohol	*E. coli*	flask	resin	105	[38]
		bioreactor	n-dodecane	87	[39]
linalool	*E. coli*	flask	none	63	[40]
		flask	n-nonane	1054	[41]
		flask	n-dodecane	505	[42]
		flask	isopropyl myristate	1250	[43]
		bioreactor	isopropyl myristate	1523.2	[44]
	S. cerevisiae	flask	none	0.095	[45]
		bioreactor	none	0.26	[46]
		bioreactor	none	23.45	[47]
		bioreactor	none	0.24	[46]
		flask	isopropyl myristate	53.14	[48]
		flask	isopropyl myristate	80.9	[49]
	Y. lipolytica	flask	n-dodecane	6.96	[50]
		flask	isopropyl myristate	109.6	[51]
	P. ananatis	tube	isopropyl myristate	5600	[52]
		bioreactor	isopropyl myristate	10900	[53]
cineole	*E. coli*	flask	n-dodecane	21	[54]
		flask	n-dodecane	653	[42]
		flask	n-nonane	116.8	[55]
	S. cerevisiae	bioreactor	none	1100	[56]
strictosidine	*S. cerevisiae*	flask	none	0.5	[57]
		bioreactor	none	50	[58]
sabinene	*E. coli*	flask	none	150	[59]
		bioreactor	none	2650	[60]
	S. cerevisiae	flask	n-dodecane	17.5	[61]
pinene	*E. coli*	bioreactor	none	970	[62]
		flask	n-dodecane	166.5	[63]
		flask	n-dodecane	140	[59]
		flask	n-dodecane	104.6	[64]
	S. cerevisiae	flask	isopropyl myristate	11.7	[65]
	C. glycerinogenes	flask	n-dodecane	6	[66]
myrcene	*E. coli*	flask	n-dodecane	58.19	[67]
		flask	isopropyl myristate	1250	[43]

Abbreviation of microorganisms: *E. coli: Escherichia coli; S. cerevisiae: Saccharomyces cerevisiae*; *C. glutamicum:*

Corynebacterium glutamicum; R. toruloides: Rhodotorula toruloides; A. gossypii: Ashbya gossypii; Y. lipolytica: Yarrowia lipolytica; P. ananatis: Pantoea ananatis; C. glycerinogenes: Candida glycerinogenes

Table S2. Summary of the fermentation results for sesquiterpenes, categorized by various chassis cells, fermentation types, the second phase, and production outputs.

Sesquiterpenes	Chassis cells	Fermentation types	Second phases	Titers (mg/L)	References
amorphadiene	*E. coli*	flask	none	112.2	[68]
		tube	n-dodecane	180	[69]
		flask	n-dodecane	201	[70]
		flask	n-dodecane	290	[71]
		flask	n-dodecane	293	[72]
		tube	n-dodecane	300	[73]
		flask	n-dodecane	331.7	[74]
		flask	n-dodecane	404.8	[75]
		flask	n-dodecane	500	[76]
		flask	n-dodecane	700	[77]
		flask	n-dodecane	1400	[78]
		bioreactor	n-dodecane	500	[79]
		bioreactor	n-dodecane	3550	[80]
		bioreactor	n-dodecane	29700	[81]
		bioreactor	n-dodecane	30000	[82]
	S. cerevisiae	flask	n-dodecane	0.6	[83]
		flask	n-dodecane	54.6	[84]
		flask	n-dodecane	64	[73]
		flask	n-dodecane	120	[85]
		flask	n-dodecane	153	[86]
		flask	n-dodecane	497	[87]
		bioreactor	n-dodecane	41000	[88]
		flask	isopropyl myristate	4000	[89]
		bioreactor	methyl oleate	40000	[89]
		bioreactor	methyl oleate	25000	[90]
	Y. lipolytica	flask	n-dodecane	171.5	[91]
	R. toruloides	bioreactor	n-dodecane	36	[92]
	B. subtilis	flask	n-dodecane	20	[93]
	S. elongatus	flask	n-hexan/n-decane	19.8	[94]
α-farnesene	*E. coli*	flask	n-decane	380	[95]
		flask	n-decane	1100	[96]
	S. cerevisiae	bioreactor	n-dodecane	23.37	[97]
		flask	n-dodecane	1477.2	[98]

		bioreactor	n-dodecane	930	[99]
		bioreactor	n-dodecane	170	[100]
		bioreactor	n-dodecane	10400	[98]
		bioreactor	poly alpha olefins	130000	[84]
	Y. lipolytica	flask	n-dodecane	1700	[101]
		bioreactor	n-dodecane	260	[102]
		bioreactor	n-dodecane	10200	[103]
		bioreactor	n-dodecane	2570	[104]
		bioreactor	n-dodecane	25550	[101]
	S. elongatus	flask	n-dodecane	4.6	[105]
		Not mentioned	n-dodecane	1.2	[106]
	P. pastoris	flask	n-dodecane	2560	[107]
	Anabaena sp.	flask	resin	0.3054	[108]
β-farnesene	*E. coli*	flask	n-decane	5290	[109]
		bioreactor	n-decane	10310	[110]
		bioreactor	n-decane	8740	[111]
		bioreactor	n-decane	4060	[112]
	S. cerevisiae	bioreactor	poly alpha olefins	130000	[84]
	Y. lipolytica	glass tubes	n-dodecane	955	[113]
		bioreactor	n-decane	22800	[114]
bisabolene	*E. coli*	tube	n-dodecane	1100	[115]
		flask	n-dodecane	1150	[116]
		flask	n-dodecane	0.435	[117]
		bioreactor	n-dodecane	900	[118]
		bioreactor	canola oil	9100	[119]
	S. cerevisiae	flask	n-dodecane	900	[118]
		flask	n-dodecane	994	[118]
		bioreactor	n-dodecane	5200	[88]
	Synechococcus sp.	flask	n-dodecane	0.6	[35]
		bioreactor	n-dodecane	22.5	[120]
	C. reinhardtii	flask	n-dodecane	11	[121]
	R. toruloides	bioreactor	n-dodecane	680	[92]
nerolidol	*E. coli*	bioreactor	n-dodecane	16000	[122]
	S. cerevisiae	flask	n-dodecane	100	[123]
		flask	n-dodecane	497	[124]
		flask	n-dodecane	392	[125]
		bioreactor	n-dodecane	1711	[126]
		bioreactor	n-dodecane	5500	[125]
		bioreactor	n-dodecane	7010	[127]
α-humulene	*E. coli*	bioreactor	resin	60.2	[128]

		bioreactor	n-dodecane	958	[129]
	S. cerevisiae	bioreactor	n-dodecane	1726.78	[130]
patchoulol	*E. coli*	bioreactor	n-dodecane	970	[131]
		bioreactor	isooctane	40	[132]
	S. cerevisiae	flask	n-dodecane	42.1	[133]
		bioreactor	n-dodecane	467	[134]
		bioreactor	n-dodecane	1632	[135]
valencene	*S. cerevisiae*	flask	n-dodecane	31	[136]
		flask	n-dodecane	1.305	[137]
		bioreactor	n-dodecane	264.6	[138]
	Y. lipolytica	flask	n-dodecane	22.8	[139]
	C. glutamicum	flask	n-dodecane	2.41	[140]
	R. sphaeroides	flask	n-dodecane	352	[141]
	Synechocystis sp.	flask	isopropyl myristate	9.6	[142]
germacrene A	*E. coli*	flask	none	6.325	[143]
		flask	n-dodecane	126.4	[144]
		flask	n-dodecane	364.26	[145]
		bioreactor	n-dodecane	3520	[146]
	S. cerevisiae	flask	n-dodecane	375	[147]
		flask	n-dodecane	190	[148]
		flask	n-dodecane	469	[149]
		flask	n-dodecane	309.8	[150]
	P. pastoris	bioreactor	n-dodecane	1900	[151]
	O. polymorpha	bioreactor	n-dodecane	4700	[152]
	Y. lipolytica	bioreactor	isopropyl myristate	39000	[153]
α-santalene	*E. coli*	bioreactor	isopropyl myristate	2916	[154]
	S. cerevisiae	flask	n-dodecane	92	[155]
		bioreactor	n-dodecane	163	[156]
	Y. lipolytica	bioreactor	n-dodecane	27.92	[157]
β-caryophyllene	*E. coli*	flask	none	100	[158]
		bioreactor	none	1520	[159]
		bioreactor	none	1050	[160]
		bioreactor	n-dodecane	5142	[161]
	S. cerevisiae	bioreactor	n-dodecane	2949.1	[162]
α-cuprenene	*X. dendrorhous*	flask	n-dodecane	80	[163]
viridiflorol	*E. coli*	bioreactor	n-dodecane	25700	[82]
longifolene	*E. coli*	bioreactor	n-decane	382	[164]
(+)-zizaene	*E. coli*	bioreactor	resin	211	[165]
valerenadiene	*E. coli*	flask	n-decane	62	[166]
protoilludene	*E. coli*	flask	n-decane	1199	[167]
farnesol	*E. coli*	flask	methyl oleate	1419	[168]

	S. cerevisiae	flask	none	70	[169]
epi-isozizaene	*E. coli*	bioreactor	n-decane	727.9	[170]
α-isocomene	*E. coli*	bioreactor	n-decane	77.5	[170]
pentalenene	*E. coli*	bioreactor	n-decane	780.3	[170]
α-neoclovene	*S. cerevisiae*	bioreactor	n-dodecane	487.1	[162]
valerenic acid	*S. cerevisiae*	flask	n-dodecane	4	[171]
zerumbone	*S. cerevisiae*	bioreactor	n-dodecane	40	[172]
prespatane	*R. toruloides*	bioreactor	n-dodecane	1173.6	[173]
santalols	*S. cerevisiae*	bioreactor	n-dodecane	1300	[174]
α-santalol	*S. cerevisiae*	bioreactor	n-dodecane	1200	[174]
zerumbone	*S. cerevisiae*	bioreactor	n-dodecane	40	[172]

Abbreviation of microorganisms*: B. subtilis: Bacillus subtilis; S. elongatus: Synechococcus elongatus; P. pastoris: Pichia pastoris; C. reinhardtii: Chlamydomonas reinhardtii; R. sphaeroides: Rhodobacter sphaeroides; O. polymorpha: Ogataea polymorpha; X. dendrorhous: Xanthophyllomyces dendrorhous*

Table S3. Summary of the fermentation results for diterpenes, categorized by various chassis cells, fermentation types, the second phase, and production outputs.

Diterpenes	Chassis cells	Fermentation types	Second phases	Titers (mg/L)	References
miltiradiene	*S. cerevisiae*	bioreactor	none	488	[175]
		flask	n-dodecane	550	[176]
		bioreactor	n-dodecane	365	[177]
		bioreactor	n-dodecane	3500	[176]
gibberellic acid 3	*Y. lipolytica*	24-roundwell plates	none	12.8	[178]
gibberellic acid 4	*Y. lipolytica*	24-roundwell plates	none	17.3	[178]
taxadiene	*E. coli*	flask	none	1.3	[179]
		flask	n-dodecane	570	[180]
		bioreactor	n-dodecane	1000	[181]
		bioreactor	n-dodecane	1020	[181]
	S. cerevisiae	bioreactor	none	33	[182]
		flask	silica gel	8	[183]
		bioreactor	n-dodecane	129	[184]
		bioreactor	n-dodecane	127	[184]
	A. fumigatus	flask	immobilization	0.694	[185]
	A. tenuissima	flask	immobilization	0.388	[185]
oxygenated taxane	*S. cerevisiae*	bioreactor	n-dodecane	78	[186]
ent -kaurene	*E. coli*	bioreactor	none	578	[187]
		bioreactor	n-dodecane	624	[188]
	R. toruloides	bioreactor	n-dodecane	1400	[189]
geranylgeraniol	*S. cerevisiae*	bioreactor	none	3300	[190]
		flask	n-dodecane	374.02	[191]
		flask	n-dodecane	772.98	[191]
		bioreactor	n-dodecane	1310	[192]

		bioreactor	n-dodecane	5070	[191]
steviol	*E. coli*	bioreactor	none	1100	[193]
		bioreactor	n-dodecane	38.4	[188]
carnosic acid	*S. cerevisiae*	flask	none	25	[194]
		bioreactor	none	75.2	[194]
sclareol	*E. coli*	bioreactor	n-dodecane	1500	[195]
	S. cerevisiae	flask	n-dodecane	750	[196]
		flask	n-dodecane	403	[197]
		bioreactor	n-hexane	11400	[198]
levopimaradiene	*E. coli*	bioreactor	n-dodecane	700	[199]
levopimaric acid	*S. cerevisiae*	bioreactor	n-dodecane	400.3	[200]
rubusoside	*S. cerevisiae*	bioreactor	none	1400	[201]
		bioreactor	none	1369	[201]
rebaudiosides	*S. cerevisiae*	bioreactor	none	132.7	[201]
retinoids	*E. coli*	tube	n-dodecane	33	[202]
retinol	*S. cerevisiae*	bioreactor	n-dodecane	2349	[203]
	Y. lipolytica	bioreactor	n-dodecane	4860	[204]
13*R*-manoyl oxide	*S. cerevisiae*	bioreactor	n-dodecane	3000	[205]
forskolin	*S. cerevisiae*	flask	n-hexane	40	[206]
cis-abienol	*E. coli*	bioreactor	isopropyl myristate	634	[207]

Abbreviation of microorganisms*: A. fumigatus: Aspergillus fumigatus; A. tenuissima: Alternaria tenuissima*

Table S4. Summary of the fermentation results for triterpenes, categorized by various chassis cells, fermentation types, the second phase, and production outputs.

Triterpenes	Chassis cells	Fermentation types	Second phases	Titers (mg/L)	References
squalene	*S. cerevisiae*	bioreactor	none	445.6	[208]
		bioreactor	none	9472	[209]
		bioreactor	n-dodecane	207.02	[98]
ambrein	*E. coli*	flask	none	2.6	[98]
	P. pastoris	bioreactor	none	100	[210]
betulin	*S. cerevisiae*	flask	none	59.5	[211]
gypsogenin	*S. cerevisiae*	bioreactor	none	146.84	[212]
lupeol	*S. cerevisiae*	flask	none	200.1	[213]
		flask	none	23.6	[211]
α-amyrin	*S. cerevisiae*	flask	none	213.7	[214]
		bioreactor	none	1100	[214]
		bioreactor	none	175.15	[215]
		flask	none	11.97	[216]
β-Amyrin	*S. cerevisiae*	bioreactor	none	138.8	[217]
		bioreactor	none	108	[218]
		tube	none	6	[219]
		bioreactor	none	108.1	[220]

		bioreactor	none	44.92	[215]
ursolic acid	*S. cerevisiae*	flask	none	101.4	[221]
		flask	none	41.4	[221]
		bioreactor	none	62.5	[221]
		bioreactor	none	123.27	[222]
betulinic acid	*S. cerevisiae*	flask	none	91.6	[221]
		flask	none	16.5	[221]
		bioreactor	none	182	[223]
		bioreactor	none	1000	[211]
		bioreactor	none	26.7	[221]
	Y. lipolytica	flask	isopropyl myristate	51.87	[224]
morolic acid	*S. cerevisiae*	flask	none	68.3	[221]
		flask	none	24.3	[221]
		bioreactor	none	34.1	[221]
		flask	none	20.7	[225]
oleanolic acid	*S. cerevisiae*	bioreactor	none	606.9	[226]
	S. cerevisiae	flask	none	186.1	[226]
	S. cerevisiae	flask	none	21.4	[227]
	S. cerevisiae	bioreactor	none	155.58	[222]
ganoderic acid	*S. cerevisiae*	flask	none	14.5	[228]
maslinic acid	*S. cerevisiae*	bioreactor	none	384	[229]
corosolic acid	*S. cerevisiae*	bioreactor	none	141	[229]
alphitolic acid	*S. cerevisiae*	bioreactor	none	23	[229]
quillaic acid	*S. cerevisiae*	bioreactor	none	314.01	[212]
polpunonic acid	*S. cerevisiae*	tube	none	1.4	[230]
glycyrrhetinic acid	*S. cerevisiae*	bioreactor	none	18.9	[220]
dammarenediol-II	*S. cerevisiae*	bioreactor	none	15000	[231]
		flask	none	211.52	[232]
		bioreactor	none	8088.8	[233]
		bioreactor	methyl oleate/n-dodecane	1548	[234]
	E. coli	flask	none	8.63	[235]
protopanaxadiol	*S. cerevisiae*	flask	none	17.2	[227]
		bioreactor	none	235	[231]
		bioreactor	methyl oleate/n-dodecane	1189	[234]
		bioreactor	none	9054.5	[233]
		bioreactor	none	1436.6	[236]
protopanaxatriol	*S. cerevisiae*	flask	none	15.9	[227]
ginsenoside Rh2	*S. cerevisiae*	bioreactor	none	300	[237]
		flask	none	16.9	[238]
		bioreactor	none	2250	[233]
ginsenoside Rg3	*S. cerevisiae*	bioreactor	none	1.3	[239]

		flask	none	51.8	[238]
ginsenoside RF1	*S. cerevisiae*	flask	none	42.1	[240]
ginsenoside Rh1	*S. cerevisiae*	flask	none	92.8	[240]

Table S5. Summary of the fermentation results for tetraterpene, categorized by various chassis cells, fermentation types, the second phase, and production outputs.

Tetraterpenes	Chassis cells	Fermentation types	Second phases	Titers (mg/L)	References
β-carotene	*E. coli*	flask	none	503	[241]
		flask	none	464	[242]
		bioreactor	none	2100	[243]
		bioreactor	none	663	[244]
		bioreactor	none	3200	[245]
		bioreactor	none	390	[246]
	S. cerevisiae	tube	none	477.9	[247]
lycopene	*E. coli*	tube	none	77.85	[248]
		flask	none	3520	[248]
		flask	none	1440	[249]
		bioreactor	none	128	[250]
		flask	none	358.9	[251]
		flask	none	224	[252]
		bioreactor	none	2700	[253]
	S. cerevisiae	flask	none	2300	[254]
		bioreactor	none	2370	[255]
		bioreactor	none	1610	[256]
	Y. lipolytica	bioreactor	none	4200	[257]
	Mucor circinelloides	flask	none	54000	[258]
	R. rubrum	flask	none	15	[259]
	R. sphaeroides	flask	none	66.05	[260]
	H. mediterranei	flask	none	429.41	[261]
	P. pastoris	bioreactor	none	73.9	[262]
		flask	none	714	[263]
astaxanthin	*E. coli*	bioreactor	none	880	[264]
		bioreactor	none	1820	[265]
crocetin	*S. cerevisiae*	bioreactor	none	6.278	[266]
zeaxanthin	*E. coli*	flask	none	43.46	[267]
		bioreactor	none	722.46	[268]
	S. cerevisiae	tube	none	1.5	[269]
	P. putida	flask	none	51.3	[270]

Abbreviation of microorganisms*: R. rubrum: Rhodospirillum rubrum; H. mediterranei: Haloferax mediterranei; P. putida:*

Pseudomonas putida

References

1. Liu, W., et al., *Engineering Escherichia coli for high-yield geraniol production with biotransformation of geranyl acetate to geraniol under fed-batch culture.* Biotechnology for Biofuels, 2016. **9**(1).
2. Xiao, L., et al., *Co-localizing key pathway enzymes by protein scaffold to enhance geraniol production in Escherichia coli.* Industrial Crops and Products, 2023. **203**.
3. Wang, X., et al., *Combined bioderivatization and engineering approach to improve the efficiency of geraniol production.* Green Chemistry, 2022. **24**(2): p. 864-876.
4. Zhou, J., et al., *Engineering Escherichia coli for selective geraniol production with minimized endogenous dehydrogenation.* Journal of Biotechnology, 2014. **169**: p. 42-50.
5. Zhou, J., et al., *Geranyl diphosphate synthase: An important regulation point in balancing a recombinant monoterpene pathway in Escherichia coli.* Enzyme and Microbial Technology, 2015. **68**: p. 50-55.
6. Tashiro, M., et al., *Directed evolution and expression tuning of geraniol synthase for efficient geraniol production in Escherichia coli.* The Journal of General and Applied Microbiology, 2017. **63**(5): p. 287-295.
7. Fischer, M.J.C., et al., *Metabolic engineering of monoterpene synthesis in yeast.* Biotechnology and Bioengineering, 2011. **108**(8): p. 1883-1892.
8. Liu, J., et al., *Overproduction of geraniol by enhanced precursor supply in Saccharomyces cerevisiae.* Journal of Biotechnology, 2013. **168**(4): p. 446-451.
9. Yee, D.A., et al., *Engineered mitochondrial production of monoterpenes in Saccharomyces cerevisiae.* Metabolic Engineering, 2019. **55**: p. 76-84.
10. Zhao, J., et al., *Improving monoterpene geraniol production through geranyl diphosphate synthesis regulation in Saccharomyces cerevisiae.* Applied Microbiology and Biotechnology, 2016. **100**: p. 4561 - 4571.
11. Zhao, J., et al., *Dynamic control of ERG20 expression combined with minimized endogenous downstream metabolism contributes to the improvement of geraniol production in Saccharomyces cerevisiae.* Microbial Cell Factories, 2017. **16**.
12. Jiang, G.-Z., et al., *Manipulation of GES and ERG20 for geraniol overproduction in Saccharomyces cerevisiae.* Metabolic Engineering, 2017. **41**: p. 57-66.
13. Li, M., S. Xu, and W. Lu, *Engineering Corynebacterium glutamicum for Geraniol Production.* Transactions of Tianjin University, 2020. **27**(5): p. 377-384.
14. Alonso-Gutierrez, J., et al., *Metabolic engineering of Escherichia coli for limonene and perillyl alcohol production.* Metabolic Engineering, 2013. **19**: p. 33-41.
15. Alonso-Gutierrez, J., et al., *Principal component analysis of proteomics (PCAP) as a tool to direct metabolic engineering.* Metabolic Engineering, 2015. **28**: p. 123-133.
16. Mendez-Perez, D., et al., *Production of jet fuel precursor monoterpenoids from engineered Escherichia coli.* Biotechnology and Bioengineering, 2017. **114**(8): p. 1703-1712.
17. Wu, J., et al., *Systematic Optimization of Limonene Production in Engineered Escherichia coli.* Journal of Agricultural and Food Chemistry, 2019. **67**(25): p. 7087-7097.
18. Willrodt, C., et al., *Decoupling production from growth by magnesium sulfate limitation boosts de novo limonene production.* Biotechnology and Bioengineering, 2015. **113**(6): p. 1305-1314.
19. Willrodt, C., et al., *Engineering the productivity of recombinant Escherichia coli for limonene formation from glycerol in minimal media.* Biotechnology Journal, 2014. **9**(8): p. 1000-1012.
20. Rolf, J., et al., *A Gram-Scale Limonene Production Process with Engineered Escherichia coli.* Molecules, 2020. **25**(8): p. 12.
21. Hu, Z., et al., *Improve the production of D-limonene by regulating the mevalonate pathway of Saccharomyces cerevisiae during alcoholic beverage fermentation.* Journal of industrial microbiology & biotechnology, 2020.

22. Jongedijk, E., et al., *Capturing of the monoterpene olefin limonene produced in Saccharomyces cerevisiae.* Yeast, 2014. **32**: p. 159 - 171.
23. Behrendorff, J.B.Y.H., et al., *2,2-Diphenyl-1-picrylhydrazyl as a screening tool for recombinant monoterpene biosynthesis.* Microbial Cell Factories, 2013. **12**: p. 76 - 76.
24. Dusséaux, S., et al., *Transforming yeast peroxisomes into microfactories for the efficient production of high-value isoprenoids.* Proceedings of the National Academy of Sciences, 2020. **117**: p. 31789 - 31799.
25. Ignea, C., et al., *Orthogonal monoterpenoid biosynthesis in yeast constructed on an isomeric substrate.* Nature Communications, 2019. **10**.
26. Peng, B., et al., *Engineered protein degradation of farnesyl pyrophosphate synthase is an effective regulatory mechanism to increase monoterpene production in Saccharomyces cerevisiae.* Metabolic engineering, 2018. **47**: p. 83-93.
27. Kong, X., et al., *Efficient Synthesis of Limonene in Saccharomyces cerevisiae Using Combinatorial Metabolic Engineering Strategies.* Journal of agricultural and food chemistry, 2023.
28. Cheng, S.J., et al., *Orthogonal Engineering of Biosynthetic Pathway for Efficient Production of Limonene in Saccharomyces cerevisiae.* ACS synthetic biology, 2019. **8 5**: p. 968-975.
29. Zhang, X., et al., *Combinatorial engineering of Saccharomyces cerevisiae for improving limonene production.* Biochemical Engineering Journal, 2021. **176**: p. 108155.
30. Liu, S., et al., *Engineering Rhodosporidium toruloides for limonene production.* Biotechnology for Biofuels, 2021. **14**.
31. Zhao, D., et al., *Optimization of Fermentation Conditions for Elevating Limonene Production with Engineered Rhodosporidium toruloides.* Fermentation, 2023.
32. Muñoz-Fernández, G., et al., *Metabolic engineering of Ashbya gossypii for limonene production from xylose.* Biotechnology for Biofuels and Bioproducts, 2022. **15**.
33. Cao, X.H., et al., *Metabolic engineering of oleaginous yeast Yarrowia lipolytica for limonene overproduction.* Biotechnology for Biofuels, 2016. **9**.
34. Cheng, B., et al., *Elevating Limonene Production in Oleaginous Yeast Yarrowia lipolytica via Genetic Engineering of Limonene Biosynthesis Pathway and Optimization of Medium Composition.* Biotechnology and Bioprocess Engineering, 2019. **24**: p. 500 - 506.
35. Davies, F., et al., *Engineering Limonene and Bisabolene Production in Wild Type and a Glycogen-Deficient Mutant of Synechococcus sp. PCC 7002.* Frontiers in Bioengineering and Biotechnology, 2014. **2**.
36. Lin, P.-C., et al., *Metabolic engineering of the pentose phosphate pathway for enhanced limonene production in the cyanobacterium Synechocystis sp. PCC 6803.* Scientific Reports, 2017. **7**.
37. Lin, P.-C., F. Zhang, and H.B. Pakrasi, *Enhanced limonene production in a fast-growing cyanobacterium through combinatorial metabolic engineering.* Metabolic Engineering Communications, 2021. **12**.
38. Alonso-Gutiérrez, J., et al., *Metabolic engineering of Escherichia coli for limonene and perillyl alcohol production.* Metabolic engineering, 2013. **19**: p. 33-41.
39. Sun, C., et al., *Effectiveness of recombinant Escherichia coli on the production of (R)-(+)-perillyl alcohol.* BMC Biotechnology, 2020. **21**.
40. Kong, S., et al., *De novo biosynthesis of linalool from glucose in engineered Escherichia coli.* Enzyme and microbial technology, 2020. **140**: p. 109614.
41. Ferraz, C.A., et al., *Isopentenol Utilization Pathway for the Production of Linalool in Escherichia coli Using an Improved Bacterial Linalool/Nerolidol Synthase.* Chembiochem, 2021. **22**: p. 2325 - 2334.
42. Mendez-Perez, D., et al., *Production of jet fuel precursor monoterpenoids from engineered Escherichia coli.* Biotechnology and Bioengineering, 2017. **114**.
43. Wang, X., et al., *Efficient Myrcene Production Using Linalool Dehydratase Isomerase and Rational Biochemical Process in Escherichia coli.* Journal of biotechnology, 2023.

44. Wu, J., et al., *Synthetic Protein Scaffolds for Improving R-(-)-Linalool Production in Escherichia coli.* Journal of agricultural and food chemistry, 2021.
45. Amiri, P., et al., *Metabolic engineering of Saccharomyces cerevisiae for linalool production.* Biotechnology Letters, 2015. **38**: p. 503 - 508.
46. Deng, Y., et al., *Enhanced (S)-linalool production by fusion expression of farnesyl diphosphate synthase and linalool synthase in Saccharomyces cerevisiae.* Journal of Applied Microbiology, 2016. **121**.
47. Zhang, Y.C., et al., *High-level production of linalool by engineered Saccharomyces cerevisiae harboring dual mevalonate pathways in mitochondria and cytoplasm.* Enzyme and microbial technology, 2020. **134**: p. 109462.
48. Zhou, P., et al., *Improved linalool production in Saccharomyces cerevisiae by combining directed evolution of linalool synthase and overexpression of the complete mevalonate pathway.* Biochemical Engineering Journal, 2020. **161**: p. 107655.
49. Zhou, P., et al., *Combinatorial Modulation of Linalool Synthase and Farnesyl Diphosphate Synthase for Linalool Overproduction in Saccharomyces cerevisiae.* Journal of agricultural and food chemistry, 2021.
50. Cao, X.H., et al., *Enhancing linalool production by engineering oleaginous yeast Yarrowia lipolytica.* Bioresource technology, 2017. **245 Pt B**: p. 1641-1644.
51. Taratynova, M.O., et al., *Boosting Geranyl Diphosphate Synthesis for Linalool Production in Engineered Yarrowia lipolytica.* Applied biochemistry and biotechnology, 2023.
52. Hoshino, Y., et al., *Stereospecific linalool production utilizing two-phase cultivation system in Pantoea ananatis.* Journal of biotechnology, 2020. **324**: p. 21-27.
53. Nitta, N., et al., *Fermentative production of enantiopure (S)-linalool using a metabolically engineered Pantoea ananatis.* Microbial Cell Factories, 2021. **20**.
54. Shaw, J.J., et al., *Identification of a Fungal 1,8-Cineole Synthase from Hypoxylon sp. with Specificity Determinants in Common with the Plant Synthases*.* The Journal of Biological Chemistry, 2015. **290**: p. 8511 - 8526.
55. Karuppiah, V., et al., *Structural Basis of Catalysis in the Bacterial Monoterpene Synthases Linalool Synthase and 1,8-Cineole Synthase.* ACS Catalysis, 2017. **7**: p. 6268 - 6282.
56. Ignea, C., et al., *Improving yeast strains using recyclable integration cassettes, for the production of plant terpenoids.* Microbial Cell Factories, 2011. **10**: p. 4 - 4.
57. Brown, S., et al., *De novo production of the plant-derived alkaloid strictosidine in yeast.* Proceedings of the National Academy of Sciences, 2015. **112**: p. 3205 - 3210.
58. Misa, J., et al., *Engineered Production of Strictosidine and Analogues in Yeast.* ACS synthetic biology, 2022.
59. Liu, H., et al., *High titer mevalonate fermentation and its feeding as a building block for isoprenoids (isoprene and sabinene) production in engineered Escherichia coli.* Process Biochemistry, 2017. **62**: p. 1-9.
60. Zhang, H., et al., *Microbial production of sabinene—a new terpene-based precursor of advanced biofuel.* Microbial Cell Factories, 2014. **13**: p. 20 - 20.
61. Ignea, C., et al., *Engineering monoterpene production in yeast using a synthetic dominant negative geranyl diphosphate synthase.* ACS synthetic biology, 2014. **3 5**: p. 298-306.
62. Yang, J., et al., *Metabolic engineering of Escherichia coli for the biosynthesis of alpha-pinene.* Biotechnology for Biofuels, 2013. **6**: p. 60 - 60.
63. Niu, F.-X., et al., *Enhancing Production of Pinene in Escherichia coli by Using a Combination of Tolerance, Evolution, and Modular Co-culture Engineering.* Frontiers in Microbiology, 2018. **9**.
64. Bao, S.-H., D.-y. Zhang, and E. Meng, *Improving biosynthetic production of pinene through plasmid recombination elimination and pathway optimization.* Plasmid, 2019: p. 102431.
65. Chen, T., Zhang,RS, *Construction of pinene-producing artificial yeast cells.* CIESC 2019, 70(1): 179-188.
66. Ma, T., et al., *Synthesis of pinene in the industrial strain Candida glycerinogenes by modification of its mevalonate pathway.* Journal of Microbiology, 2022. **60**: p. 1191 - 1200.

67. Kim, E.-M., et al., *Microbial Synthesis of Myrcene by Metabolically Engineered Escherichia coli.* Journal of agricultural and food chemistry, 2015. **63 18**: p. 4606-12.
68. Martin, V.J.J., et al., *Engineering a mevalonate pathway in Escherichia coli for production of terpenoids.* Nature Biotechnology, 2003. **21**: p. 796-802.
69. Zhang, C., et al., *Efflux transporter engineering markedly improves amorphadiene production in Escherichia coli.* Biotechnology and Bioengineering, 2016. **113**.
70. Zhang, C., et al., *Combining Genotype Improvement and Statistical Media Optimization for Isoprenoid Production in E. coli.* PLoS ONE, 2013. **8**.
71. Pitera, D.J., et al., *Balancing a heterologous mevalonate pathway for improved isoprenoid production in Escherichia coli.* Metabolic engineering, 2007. **9 2**: p. 193-207.
72. Anthony, J.R., et al., *Optimization of the mevalonate-based isoprenoid biosynthetic pathway in Escherichia coli for production of the anti-malarial drug precursor amorpha-4,11-diene.* Metabolic engineering, 2009. **11 1**: p. 13-9.
73. Yuan, J. and C.B. Ching, *Combinatorial engineering of mevalonate pathway for improved amorpha-4,11-diene production in budding yeast.* Biotechnology and Bioengineering, 2014. **111**.
74. Wang, J.-f., et al., *Exploiting exogenous MEP pathway genes to improve the downstream isoprenoid pathway effects and enhance isoprenoid production in Escherichia coli.* Process Biochemistry, 2015. **50**: p. 24-32.
75. Wang, J.-F., et al., *Enhancing isoprenoid production through systematically assembling and modulating efflux pumps in Escherichia coli.* Applied Microbiology and Biotechnology, 2013. **97**: p. 8057-8067.
76. Redding-Johanson, A.M., et al., *Targeted proteomics for metabolic pathway optimization: application to terpene production.* Metabolic engineering, 2011. **13 2**: p. 194-203.
77. Ma, S.M., et al., *Optimization of a heterologous mevalonate pathway through the use of variant HMG-CoA reductases.* Metabolic engineering, 2011. **13 5**: p. 588-97.
78. Dahl, R.H., et al., *Engineering dynamic pathway regulation using stress-response promoters.* Nature Biotechnology, 2013. **31**: p. 1039-1046.
79. Newman, J.D., et al., *High-level production of amorpha-4,11-diene in a two-phase partitioning bioreactor of metabolically engineered Escherichia coli.* Biotechnology and Bioengineering, 2006. **95**.
80. Nowroozi, F.F., et al., *Metabolic pathway optimization using ribosome binding site variants and combinatorial gene assembly.* Applied Microbiology and Biotechnology, 2014. **98**: p. 1567-1581.
81. Tsuruta, H., et al., *High-Level Production of Amorpha-4,11-Diene, a Precursor of the Antimalarial Agent Artemisinin, in Escherichia coli.* PLoS ONE, 2009. **4**.
82. Shukal, S., X. Chen, and C. Zhang, *Systematic engineering for high-yield production of viridiflorol and amorphadiene in auxotrophic Escherichia coli.* Metabolic engineering, 2019.
83. Lindahl, A., et al., *Production of the Artemisinin Precursor Amorpha-4,11-diene by Engineered Saccharomyces cerevisiae.* Biotechnology Letters, 2006. **28**: p. 571-580.
84. Meadows, A.L., et al., *Rewriting yeast central carbon metabolism for industrial isoprenoid production.* Nature, 2016. **537**: p. 694-697.
85. Shiba, Y., et al., *Engineering of the pyruvate dehydrogenase bypass in Saccharomyces cerevisiae for high-level production of isoprenoids.* Metabolic engineering, 2007. **9 2**: p. 160-8.
86. Ro, D.-K., et al., *Production of the antimalarial drug precursor artemisinic acid in engineered yeast.* Nature, 2006. **440**: p. 940-943.
87. Kwak, S., et al., *Redirection of the Glycolytic Flux Enhances Isoprenoid Production in Saccharomyces Cerevisiae.* Biotechnology journal, 2020.
88. Özaydın, B., et al., *Carotenoid-based phenotypic screen of the yeast deletion collection reveals new genes with roles in isoprenoid production.* Metabolic engineering, 2013. **15**: p. 174-83.

89. Westfall, P., et al., *Production of amorphadiene in yeast, and its conversion to dihydroartemisinic acid, precursor to the antimalarial agent artemisinin.* Proceedings of the National Academy of Sciences, 2012. **109**: p. E111 - E118.

90. Paddon, C.J., et al., *High-level semi-synthetic production of the potent antimalarial artemisinin.* Nature, 2013. **496**: p. 528-532.

91. Marsafari, M. and P. Xu, *Debottlenecking mevalonate pathway for antimalarial drug precursor amorphadiene biosynthesis in Yarrowia lipolytica.* Metabolic Engineering Communications, 2020. **10**.

92. Yaegashi, J., et al., *Rhodosporidium toruloides: a new platform organism for conversion of lignocellulose into terpene biofuels and bioproducts.* Biotechnology for Biofuels, 2017. **10**.

93. Zhou, K., et al., *Optimization of amorphadiene synthesis in bacillus subtilis via transcriptional, translational, and media modulation.* Biotechnology and bioengineering, 2013. **110 9**: p. 2556-61.

94. Choi, S.Y., et al., *Photosynthetic conversion of CO2 to farnesyl diphosphate-derived phytochemicals (amorpha-4,11-diene and squalene) by engineered cyanobacteria.* Biotechnology for Biofuels, 2016. **9**.

95. Wang, C., et al., *Metabolic engineering of Escherichia coli for α-farnesene production.* Metabolic engineering, 2011. **13 6**: p. 648-55.

96. Zhu, F., et al., *In vitro reconstitution of mevalonate pathway and targeted engineering of farnesene overproduction in Escherichia coli.* Biotechnology and Bioengineering, 2014. **111**.

97. Yang, X., et al., *Quorum sensing-mediated protein degradation for dynamic metabolic pathway control in Saccharomyces cerevisiae.* Metabolic engineering, 2021. **64**: p. 85-94.

98. Wang, J., et al., *Overproduction of α-Farnesene in Saccharomyces cerevisiae by Farnesene Synthase Screening and Metabolic Engineering.* Journal of agricultural and food chemistry, 2021.

99. Han, J.Y., et al., *Ty1-fused protein-body formation for spatial organization of metabolic pathways in Saccharomyces cerevisiae.* Biotechnology and Bioengineering, 2018. **115**: p. 694 - 704.

100. Tippmann, S., et al., *Production of farnesene and santalene by Saccharomyces cerevisiae using fed-batch cultivations with RQ-controlled feed.* Biotechnology and bioengineering, 2016. **113 1**: p. 72-81.

101. Liu, Y., et al., *Engineering the oleaginous yeast Yarrowia lipolytica for production of α-farnesene.* Biotechnology for Biofuels, 2019. **12**.

102. Yang, X., et al., *Heterologous production of α-farnesene in metabolically engineered strains of Yarrowia lipolytica.* Bioresource technology, 2016. **216**: p. 1040-8.

103. Liu, Y., et al., *α-Farnesene production from lipid by engineered Yarrowia lipolytica.* Bioresources and Bioprocessing, 2021. **8**: p. 1-12.

104. Liu, S.-C., et al., *Pathway engineering and medium optimization for α-farnesene biosynthesis in oleaginous yeast Yarrowia lipolytica.* Journal of biotechnology, 2020.

105. Lee, H.J., et al., *Direct Conversion of CO2 to α-Farnesene Using Metabolically Engineered Synechococcus elongatus PCC 7942.* Journal of agricultural and food chemistry, 2017. **65 48**: p. 10424-10428.

106. Pattharaprachayakul, N., et al., *Evolutionary engineering of cyanobacteria to enhance the production of α-farnesene from CO2.* Journal of agricultural and food chemistry, 2019.

107. Liu, H., et al., *Dual Regulation of Cytoplasm and Peroxisomes for Improved A-Farnesene Production in Recombinant Pichia pastoris.* ACS synthetic biology, 2021.

108. Halfmann, C.T., et al., *Genetically engineering cyanobacteria to convert CO2, water, and light into the long-chain hydrocarbon farnesene.* Applied Microbiology and Biotechnology, 2014. **98**: p. 9869 - 9877.

109. Lv, J., et al., *Highly efficient production of FAMEs and β-farnesene from a two-stage biotransformation of waste cooking oils.* Energy Conversion and Management, 2019.

110. Yao, P., et al., *Investigation of fermentation conditions of biodiesel by-products for high production of β-farnesene by an engineered Escherichia coli.* Environmental Science and Pollution Research, 2020. **27**: p. 22758 - 22769.

111. You, S., et al., *Utilization of biodiesel by-product as substrate for high-production of β-farnesene via relatively balanced mevalonate pathway in Escherichia coli.* Bioresource technology, 2017. **243**: p. 228-236.
112. You, S., et al., *Recycling strategy and repression elimination for lignocellulosic-based farnesene production with an engineered Escherichia coli.* Journal of agricultural and food chemistry, 2019.
113. Arnesen, J.A., et al., *Yarrowia lipolytica Strains Engineered for the Production of Terpenoids.* Frontiers in Bioengineering and Biotechnology, 2020. **8**.
114. Shi, T., et al., *Engineering the oleaginous yeast Yarrowia lipolytica for β-farnesene overproduction.* Biotechnology Journal, 2021. **16**.
115. Kim, E.-M., et al., *Autonomous control of metabolic state by a quorum sensing (QS)-mediated regulator for bisabolene production in engineered E. coli.* Metabolic engineering, 2017. **44**: p. 325-336.
116. Alonso-Gutiérrez, J., et al., *Principal component analysis of proteomics (PCAP) as a tool to direct metabolic engineering.* Metabolic engineering, 2015. **28**: p. 123-133.
117. Alonso-Gutiérrez, J., et al., *Toward industrial production of isoprenoids in Escherichia coli: Lessons learned from CRISPR-Cas9 based optimization of a chromosomally integrated mevalonate pathway.* Biotechnology and Bioengineering, 2018. **115**: p. 1000 - 1013.
118. Peralta-Yahya, P., et al., *Identification and microbial production of a terpene-based advanced biofuel.* Nature Communications, 2011. **2**.
119. Han, G.H., et al., *Fermentative production and direct extraction of (−)-α-bisabolol in metabolically engineered Escherichia coli.* Microbial Cell Factories, 2016. **15**.
120. Sebesta, J. and C.A.M. Peebles, *Improving heterologous protein expression in Synechocystis sp. PCC 6803 for alpha-bisabolene production.* Metabolic Engineering Communications, 2019. **10**.
121. Wichmann, J., et al., *Tailored carbon partitioning for phototrophic production of (E)-α-bisabolene from the green microalga Chlamydomonas reinhardtii.* Metabolic engineering, 2018. **45**: p. 211-222.
122. Tan, N., et al., *High-Yield Biosynthesis of trans-Nerolidol from Sugar and Glycerol.* Journal of agricultural and food chemistry, 2023.
123. Peng, B., et al., *A squalene synthase protein degradation method for improved sesquiterpene production in Saccharomyces cerevisiae.* Metabolic engineering, 2017. **39**: p. 209-219.
124. Qu, Z., et al., *Overexpression of the transcription factor HAC1 improves nerolidol production in engineered yeast.* Enzyme and microbial technology, 2020. **134**: p. 109485.
125. Peng, B., et al., *Coupling gene regulatory patterns to bioprocess conditions to optimize synthetic metabolic modules for improved sesquiterpene production in yeast.* Biotechnology for Biofuels, 2017. **10**.
126. Zhang, L., et al., *Creation of a yeast cell factory for high production of nerol tertiary alcohols.* China Journal of Chinese Materia Medica, 2017. **42**(15): p. 7.
127. Li, W.-g., et al., *Characterization of trans-Nerolidol Synthase from Celastrus angulatus Maxim and Production of trans-Nerolidol in Engineered Saccharomyces cerevisiae.* Journal of agricultural and food chemistry, 2021.
128. Alemdar, S., et al., *Bioproduction of α-humulene in metabolically engineered Escherichia coli and application in zerumbone synthesis.* Engineering in Life Sciences, 2017. **17**.
129. Harada, H., et al., *Efficient synthesis of functional isoprenoids from acetoacetate through metabolic pathway-engineered Escherichia coli.* Applied Microbiology and Biotechnology, 2009. **81**: p. 915-925.
130. Zhang, C., et al., *Harnessing yeast peroxisomes and cytosol acetyl-CoA for sesquiterpene α-humulene production.* Journal of agricultural and food chemistry, 2020.
131. Zhou, L., et al., *Enhancement of Patchoulol Production in Escherichia coli via Multiple Engineering Strategies.* Journal of agricultural and food chemistry, 2021.
132. Aguilar, F., et al., *Whole-Cell Production of Patchouli Oil Sesquiterpenes in Escherichia coli: Metabolic Engineering and*

Fermentation Optimization in Solid–Liquid Phase Partitioning Cultivation. ACS Omega, 2020. **5**: p. 32436 - 32446.
133. Albertsen, L., et al., *Diversion of Flux toward Sesquiterpene Production in Saccharomyces cerevisiae by Fusion of Host and Heterologous Enzymes.* Applied and Environmental Microbiology, 2010. **77**: p. 1033 - 1040.
134. Ma, B., et al., *Significantly enhanced production of patchoulol in metabolically engineered Saccharomyces cerevisiae.* Journal of agricultural and food chemistry, 2019.
135. Liu, M., et al., *High-Level Production of Sesquiterpene Patchoulol in Saccharomyces cerevisiae.* ACS synthetic biology, 2021.
136. Emmerstorfer, A., et al., *Over-expression of ICE2 stabilizes cytochrome P450 reductase in Saccharomyces cerevisiae and Pichia pastoris.* Biotechnology journal, 2015. **10 4**: p. 623-35.
137. Cankar, K., et al., *Valencene oxidase CYP706M1 from Alaska cedar (Callitropsis nootkatensis).* FEBS Letters, 2014. **588**.
138. Ouyang, X., et al., *Production of valencene and its derivatives by recombinant brewer's yeast fermentation.* Food and Fermentation Industries Editorial Staff, 2019. **45**(20): p. 9.
139. Guo, X., et al., *Heterologous biosynthesis of (+)-nootkatone in unconventional yeast Yarrowia lipolytica.* Biochemical Engineering Journal, 2018.
140. Frohwitter, J., et al., *Production of the sesquiterpene (+)-valencene by metabolically engineered Corynebacterium glutamicum.* Journal of biotechnology, 2014. **191**: p. 205-13.
141. Beekwilder, J., et al., *Valencene synthase from the heartwood of Nootka cypress (Callitropsis nootkatensis) for biotechnological production of valencene.* Plant biotechnology journal, 2014. **12 2**: p. 174-82.
142. Matsudaira, A., et al., *Production of glutamate and stereospecific flavors, (S)-linalool and (+)-valencene, by Synechocystis sp. PCC6803.* Journal of bioscience and bioengineering, 2020.
143. Gao, Y., *The study of microbial synthesis of germacrene A the precursor of β-elemene*. 2012, Hangzhou Normal University.
144. Chen, R., et al., *Protein Engineering of a Germacrene A Synthase From Lactuca sativa and Its Application in High Productivity of Germacrene A in Escherichia coli.* Frontiers in Plant Science, 2022. **13**.
145. Li, M., et al., *Co-biosynthesis of germacrene A, a precursor of β-elemene, and lycopene in engineered Escherichia coli.* Applied Microbiology and Biotechnology, 2022. **106**: p. 8053 - 8066.
146. Fordjour, E., et al., *Engineering Escherichia coli BL21 (DE3) for high-yield production of germacrene A, a precursor of β-elemene via combinatorial metabolic engineering strategies.* Biotechnology and Bioengineering, 2023. **120**: p. 3039 - 3056.
147. Bröker, J.N., et al., *Combinatorial Metabolic Engineering in Saccharomyces cerevisiae for the Enhanced Production of the FPP-Derived Sesquiterpene Germacrene.* Bioengineering, 2020. **7**.
148. *Metabolic engineering of Saccharomyces cerevisiae for production of germacrene A, a precursor of beta-elemene.* Journal of Industrial Microbiology & Biotechnology, 2017. **44**: p. 1065-1072.
149. Zhang, X., et al., *A recombinant bacterium and its use*. 2021.
150. Zhang, W., et al., *Improved production of germacrene A, a direct precursor of β-elemene, in engineered Saccharomyces cerevisiae by expressing a cyanobacterial germacrene A synthase.* Microbial Cell Factories, 2020. **20**.
151. Cheng, J., et al., *Development of a Pichia pastoris cell factory for efficient production of germacrene A: a precursor of β-elemene.* Bioresources and Bioprocessing, 2023. **10**: p. 1-9.
152. Ye, M., J. Gao, and Y.J. Zhou, *Global metabolic rewiring of the nonconventional yeast Ogataea polymorpha for biosynthesis of the sesquiterpenoid β-elemene.* Metabolic engineering, 2023.
153. Liu, Q., et al., *Reprogramming the metabolism of oleaginous yeast for sustainably biosynthesizing the anticarcinogen precursor germacrene A.* Green Chemistry, 2023.
154. Zhang, J., et al., *Sesquiterpene Synthase Engineering and Targeted Engineering of α-Santalene Overproduction in Escherichia coli.* Journal of agricultural and food chemistry, 2022.
155. Scalcinati, G., et al., *Dynamic control of gene expression in Saccharomyces cerevisiae engineered for the production of plant*

sesquitepene α-santalene in a fed-batch mode. Metabolic engineering, 2012. **14 2**: p. 91-103.
156. Asadollahi, M.A., et al., *Production of plant sesquiterpenes in Saccharomyces cerevisiae: Effect of ERG9 repression on sesquiterpene biosynthesis.* Biotechnology and Bioengineering, 2008. **99**.
157. Jia, D., et al., *Yarrowia lipolytica construction for heterologous synthesis of α-santalene and fermentation optimization.* Applied Microbiology and Biotechnology, 2019. **103**: p. 3511 - 3520.
158. Wu, W., F. Liu, and R.W. Davis, *Engineering Escherichia coli for the production of terpene mixture enriched in caryophyllene and caryophyllene alcohol as potential aviation fuel compounds.* Metabolic Engineering Communications, 2018. **6**: p. 13 - 21.
159. Yang, J., et al., *Biosynthesis of β-caryophyllene, a novel terpene-based high-density biofuel precursor, using engineered Escherichia coli.* Renewable Energy, 2016. **99**: p. 216-223.
160. Yang, J. and Q. Nie, *Engineering Escherichia coli to convert acetic acid to β-caryophyllene.* Microbial Cell Factories, 2016. **15**.
161. Cheng, T., et al., *Highly efficient biosynthesis of β-caryophyllene with a new sesquiterpene synthase from tobacco.* Biotechnology for Biofuels and Bioproducts, 2022. **15**.
162. Li, X., et al., *Production of sesquiterpenoids α-neoclovene and β-caryophyllene by engineered Saccharomyces cerevisiae.* Synthetic Biology Journal, 2021(005): p. 002.
163. Melillo, E., et al., *Production of α-cuprenene in Xanthophyllomyces dendrorhous: a step closer to a potent terpene biofactory.* Microbial Cell Factories, 2013. **12**: p. 13 - 13.
164. Cao, Y., et al., *Manipulation of the precursor supply for high-level production of longifolene by metabolically engineered Escherichia coli.* Scientific Reports, 2019. **9**.
165. Aguilar, F., T. Scheper, and S. Beutel, *Improved Production and In Situ Recovery of Sesquiterpene (+)-Zizaene from Metabolically-Engineered E. coli.* Molecules, 2019. **24**.
166. Nybo, S.E., J. Saunders, and S.P. McCormick, *Metabolic engineering of Escherichia coli for production of valerenadiene.* Journal of biotechnology, 2017. **262**: p. 60-66.
167. Yang, L., et al., *Combinatorial engineering of hybrid mevalonate pathways in Escherichiacoli for protoilludene production.* Microbial Cell Factories, 2016. **15**.
168. Zada, B., et al., *Metabolic engineering of Escherichia coli for production of mixed isoprenoid alcohols and their derivatives.* Biotechnology for Biofuels, 2018. **11**.
169. Zhuang, X. and J. Chappell, *Building terpene production platforms in yeast.* Biotechnology and Bioengineering, 2015. **112**.
170. Liu, C.-L., et al., *Renewable production of high density jet fuel precursor sesquiterpenes from Escherichia coli.* Biotechnology for Biofuels, 2018. **11**.
171. Wong, J.Y.F., et al., *De novo synthesis of the sedative valerenic acid in Saccharomyces cerevisiae.* Metabolic engineering, 2018. **47**: p. 94-101.
172. Zhang, C., et al., *Production of sesquiterpenoid zerumbone from metabolic engineered Saccharomyces cerevisiae.* Metabolic engineering, 2018. **49**: p. 28-35.
173. Geiselman, G.M., et al., *Conversion of poplar biomass into high-energy density tricyclic sesquiterpene jet fuel blendstocks.* Microbial Cell Factories, 2020. **19**.
174. Zha, W.-l., et al., *Reconstruction of the Biosynthetic Pathway of Santalols under Control of the GAL Regulatory System in Yeast.* ACS synthetic biology, 2020.
175. Dai, Z., et al., *Production of miltiradiene by metabolically engineered Saccharomyces cerevisiae.* Biotechnology and Bioengineering, 2012. **109**.
176. Hu, T., et al., *Engineering chimeric diterpene synthases and isoprenoid biosynthetic pathways enables high-level production of miltiradiene in yeast.* Metabolic engineering, 2020.
177. Zhou, Y.J., et al., *Modular pathway engineering of diterpenoid synthases and the mevalonic acid pathway for miltiradiene*

production. Journal of the American Chemical Society, 2012. **134 6**: p. 3234-41.
178. Kildegaard, K.R., et al., *Tailored biosynthesis of gibberellin plant hormones in yeast.* Metabolic Engineering, 2021. **66**: p. 1 - 11.
179. Huang, Q., et al., *Engineering Escherichia coli for the synthesis of taxadiene, a key intermediate in the biosynthesis of taxol.* Bioorganic & medicinal chemistry, 2001. **9 9**: p. 2237-42.
180. Biggs, B.W., et al., *Overcoming heterologous protein interdependency to optimize P450-mediated Taxol precursor synthesis in Escherichia coli.* Proceedings of the National Academy of Sciences, 2016. **113**: p. 3209 - 3214.
181. Ajikumar, P.K., et al., *Isoprenoid Pathway Optimization for Taxol Precursor Overproduction in Escherichia coli.* Science, 2010. **330**: p. 70 - 74.
182. Zhou, K., et al., *Distributing a metabolic pathway among a microbial consortium enhances production of natural products.* Nature biotechnology, 2015. **33**: p. 377 - 383.
183. Engels, B., P. Dahm, and S. Jennewein, *Metabolic engineering of taxadiene biosynthesis in yeast as a first step towards Taxol (Paclitaxel) production.* Metabolic engineering, 2008. **10 3-4**: p. 201-6.
184. Nowrouzi, B., et al., *Enhanced production of taxadiene in Saccharomyces cerevisiae.* Microbial Cell Factories, 2020. **19**.
185. El-Sayed, E.-S.R., et al., *Strain improvement and immobilization technique for enhanced production of the anticancer drug paclitaxel by Aspergillus fumigatus and Alternaria tenuissima.* Applied Microbiology and Biotechnology, 2019. **103**: p. 8923 - 8935.
186. Walls, L.E., et al., *Optimizing the biosynthesis of oxygenated and acetylated Taxol precursors in Saccharomyces cerevisiae using advanced bioprocessing strategies.* Biotechnology and Bioengineering, 2020. **118**: p. 279 - 293.
187. Kong, M.K., et al., *Metabolic engineering of the Stevia rebaudiana ent-kaurene biosynthetic pathway in recombinant Escherichia coli.* Journal of biotechnology, 2015. **214**: p. 95-102.
188. Moon, J.H., et al., *Redesign and reconstruction of a steviol-biosynthetic pathway for enhanced production of steviol in Escherichia coli.* Microbial Cell Factories, 2020. **19**.
189. Geiselman, G.M., et al., *Production of ent-kaurene from lignocellulosic hydrolysate in Rhodosporidium toruloides.* Microbial Cell Factories, 2020. **19**.
190. Tokuhiro, K., et al., *Overproduction of Geranylgeraniol by Metabolically Engineered Saccharomyces cerevisiae.* Applied and Environmental Microbiology, 2009. **75**: p. 5536 - 5543.
191. Wang, J., et al., *Enhancing Geranylgeraniol Production by Metabolic Engineering and Utilization of Isoprenol as a Substrate in Saccharomyces cerevisiae.* Journal of agricultural and food chemistry, 2021.
192. Song, T.-Q., et al., *Engineering Saccharomyces cerevisiae for geranylgeraniol overproduction by combinatorial design.* Scientific Reports, 2017. **7**.
193. Sun, Y., et al., *De novo production of versatile oxidized kaurene diterpenes in Escherichia coli.* Metabolic engineering, 2022.
194. Wei, P., et al., *Metabolic Engineering of Saccharomyces cerevisiae for Heterologous Carnosic Acid Production.* Frontiers in Bioengineering and Biotechnology, 2022. **10**.
195. Schalk, M., et al., *Toward a biosynthetic route to sclareol and amber odorants.* Journal of the American Chemical Society, 2012. **134 46**: p. 18900-3.
196. Ignea, C., et al., *Efficient diterpene production in yeast by engineering Erg20p into a geranylgeranyl diphosphate synthase.* Metabolic engineering, 2015. **27**: p. 65-75.
197. Ignea, C., et al., *Reconstructing the chemical diversity of labdane-type diterpene biosynthesis in yeast.* Metabolic engineering, 2015. **28**: p. 91-103.
198. Cao, X.H., et al., *Engineering yeast for high-level production of diterpenoid sclareol.* Metabolic engineering, 2022.
199. Leonard, E., et al., *Combining metabolic and protein engineering of a terpenoid biosynthetic pathway for overproduction and selectivity control.* Proceedings of the National Academy of Sciences, 2010. **107**: p. 13654 - 13659.
200. Liu, T., C. Zhang, and W. Lu, *Heterologous production of levopimaric acid in Saccharomyces cerevisiae.* Microbial Cell

Factories, 2018. **17**.
201. Xu, Y., et al., *De novo biosynthesis of rubusoside and rebaudiosides in engineered yeasts.* Nature Communications, 2022. **13**.
202. Zhang, C., et al., *A "plug-n-play" modular metabolic system for the production of apocarotenoids.* Biotechnology and Bioengineering, 2018. **115**: p. 174 - 183.
203. Hu, Q., et al., *Selective biosynthesis of retinol in S. cerevisiae.* Bioresources and Bioprocessing, 2022. **9**: p. 1-14.
204. Park, H.G., et al., *Efficient production of retinol in Yarrowia lipolytica by increasing stability using antioxidant and detergent extraction.* Metabolic engineering, 2022.
205. Zhang, C., et al., *High-titer production of 13R-manoyl oxide in metabolically engineered Saccharomyces cerevisiae.* Microbial Cell Factories, 2019. **18**.
206. Pateraki, I., et al., *Total biosynthesis of the cyclic AMP booster forskolin from Coleus forskohlii.* eLife, 2017. **6**.
207. Li, L., et al., *Combinatorial Engineering of Mevalonate Pathway and Diterpenoid Synthases in Escherichia coli for cis-Abienol Production.* Journal of agricultural and food chemistry, 2019. **67 23**: p. 6523-6531.
208. Wei, L., et al., *Improved squalene production through increasing lipid contents in Saccharomyces cerevisiae.* Biotechnology and Bioengineering, 2018. **115**: p. 1793 - 1800.
209. Li, T., et al., *Metabolic engineering of Saccharomyces cerevisiae to overproduce squalene.* Journal of agricultural and food chemistry, 2020.
210. Moser, S., et al., *Whole-cell (+)-ambrein production in the yeast Pichia pastoris.* Metabolic Engineering Communications, 2018. **7**: p. e00077 - e00077.
211. Huang, J., et al., *Identification of RoCYP01 (CYP716A155) enables construction of engineered yeast for high-yield production of betulinic acid.* Applied Microbiology and Biotechnology, 2019. **103**: p. 7029 - 7039.
212. Li, W., et al., *De Novo Biosynthesis of the Oleanane-Type Triterpenoids of Tunicosaponins in Yeast.* ACS synthetic biology, 2021.
213. Qiao, W., et al., *Improving lupeol production in yeast by recruiting pathway genes from different organisms.* Scientific Reports, 2019. **9**.
214. Yu, Y., et al., *Engineering Saccharomyces cerevisiae for high yield production of α-amyrin via synergistic remodeling of α-amyrin synthase and expanding the storage pool.* Metabolic engineering, 2020.
215. Jin, K., et al., *Combinatorial metabolic engineering enables the efficient production of ursolic acid and oleanolic acid in Saccharomyces cerevisiae.* Bioresource technology, 2023: p. 128819.
216. Yu, Y., et al., *Productive Amyrin Synthases for Efficient α-Amyrin Synthesis in Engineered Saccharomyces cerevisiae.* ACS synthetic biology, 2018. **7 10**: p. 2391-2402.
217. Zhang, G., et al., *Refactoring β-amyrin synthesis in Saccharomyces cerevisiae.* Aiche Journal, 2015. **61**: p. 3172-3179.
218. Muhammad Saad Ahmed, P.D., et al., *Design and construction of short synthetic terminators for β-amyrin production in Saccharomyces cerevisiae.* Biochemical Engineering Journal, 2019.
219. Kirby, J., et al., *Engineering triterpene production in Saccharomyces cerevisiae–β-amyrin synthase from Artemisia annua.* The FEBS Journal, 2008. **275**.
220. Zhu, M.D., et al., *Boosting 11-oxo-β-amyrin and glycyrrhetinic acid synthesis in Saccharomyces cerevisiae via pairing novel oxidation and reduction system from legume plants.* Metabolic engineering, 2018. **45**: p. 43-50.
221. Gao, H., *Construction of cell factories for production of pentacyclic triterpenoids in Saccharomyces cerevisiae.* 2021.
222. Lu, C., et al., *Biosynthesis of ursolic acid and oleanolic acid in Saccharomyces cerevisiae.* Aiche Journal, 2018. **64**: p. 3794-3802.
223. Czarnotta, E., et al., *Fermentation and purification strategies for the production of betulinic acid and its lupane-type precursors in Saccharomyces cerevisiae.* Biotechnology and Bioengineering, 2017. **114**: p. 2528 - 2538.
224. Jin, C.-C., et al., *Boosting the biosynthesis of betulinic acid and related triterpenoids in Yarrowia lipolytica via multimodular metabolic engineering.* Microbial Cell Factories, 2019. **18**.

225. Srisawat, P., et al., *Production of the bioactive plant-derived triterpenoid morolic acid in engineered Saccharomyces cerevisiae.* Biotechnology and Bioengineering, 2020. **117**: p. 2198 - 2208.

226. Zhao, Y.-j., et al., *Enhancing oleanolic acid production in engineered Saccharomyces cerevisiae.* Bioresource technology, 2018. **257**: p. 339-343.

227. Dai, Z., et al., *Producing aglycons of ginsenosides in bakers' yeast.* Scientific Reports, 2014. **4**.

228. Wang, W.-F., H. Xiao, and J.-J. Zhong, *Biosynthesis of a ganoderic acid in Saccharomyces cerevisiae by expressing a cytochrome P450 gene from Ganoderma lucidum.* Biotechnology and Bioengineering, 2018. **115**: p. 1842 - 1854.

229. Dai, Z., et al., *Identification of a novel cytochrome P450 enzyme that catalyzes the C-2α hydroxylation of pentacyclic triterpenoids and its application in yeast cell factories.* Metabolic engineering, 2019. **51**: p. 70-78.

230. Hansen, N.L., et al., *Integrating pathway elucidation with yeast engineering to produce polpunonic acid the precursor of the anti-obesity agent celastrol.* Microbial Cell Factories, 2020. **19**.

231. Wang, D., et al., *Construction of efficient yeast cell factories for production of ginsenosides precursor dammarenediol-II.* Acta Pharmaceutica Sinica B, 2018. **053**(008): p. 1233-1241.

232. zhang, x., *Performance Optimization of Saccharomyces cerevisiae for Dammarendiol-II Production* 2015, Tianjin University.

233. Wang, P., et al., *Synthesizing ginsenoside Rh2 in Saccharomyces cerevisiae cell factory at high-efficiency.* Cell Discovery, 2019. **5**.

234. Dai, Z., et al., *Metabolic engineering of Saccharomyces cerevisiae for production of ginsenosides.* Metabolic engineering, 2013. **20**: p. 146-56.

235. Li, D., et al., *Heterologous biosynthesis of triterpenoid dammarenediol-II in engineered Escherichia coli.* Biotechnology Letters, 2016. **38**: p. 603 - 609.

236. Zhao, F., et al., *Optimization of a cytochrome P450 oxidation system for enhancing protopanaxadiol production in Saccharomyces cerevisiae.* Biotechnology and Bioengineering, 2016. **113**.

237. Yu, Z., et al., *Biosynthesis of plant-derived ginsenoside Rh2 in yeast via repurposing a key promiscuous microbial enzyme.* Metabolic engineering, 2017. **42**: p. 25-32.

238. Wang, P., et al., *Production of bioactive ginsenosides Rh2 and Rg3 by metabolically engineered yeasts.* Metabolic engineering, 2015. **29**: p. 97-105.

239. Jung, S.-C., et al., *Two ginseng UDP-glycosyltransferases synthesize ginsenoside Rg3 and Rd.* Plant & cell physiology, 2014. **55 12**: p. 2177-88.

240. Wei, W., et al., *Characterization of Panax ginseng UDP-Glycosyltransferases Catalyzing Protopanaxatriol and Biosyntheses of Bioactive Ginsenosides F1 and Rh1 in Metabolically Engineered Yeasts.* Molecular plant, 2015. **8 9**: p. 1412-24.

241. Yoon, S.H., et al., *Increased β-Carotene Production in Recombinant Escherichia coli Harboring an Engineered Isoprenoid Precursor Pathway with Mevalonate Addition.* Biotechnology Progress, 2008. **23**.

242. Yoon, S.H., et al., *Combinatorial expression of bacterial whole mevalonate pathway for the production of beta-carotene in E. coli.* Journal of biotechnology, 2009. **140 3-4**: p. 218-26.

243. Zhao, J., et al., *Engineering central metabolic modules of Escherichia coli for improving β-carotene production.* Metabolic engineering, 2013. **17**: p. 42-50.

244. Kim, J.H., et al., *Production of β-carotene by recombinant Escherichia coli with engineered whole mevalonate pathway in batch and fed-batch cultures.* Biotechnology and Bioprocess Engineering, 2009. **14**: p. 559-564.

245. Yang, J. and L. Guo, *Biosynthesis of β-carotene in engineered E. coli using the MEP and MVA pathways.* Microbial Cell Factories, 2014. **13**.

246. Kim, S.-W., et al., *Over-production of beta-carotene from metabolically engineered Escherichia coli.* Biotechnology letters, 2006. **28 12**: p. 897-904.

247. Fathi, Z., et al., *Metabolic engineering of Saccharomyces cerevisiae for production of β-carotene from hydrophobic substrates.* FEMS Yeast Research, 2020. **21**.

248. Chen, Y., et al., *Chromosomal evolution of Escherichia coli for the efficient production of lycopene.* BMC Biotechnology, 2013. **13**: p. 6 - 6.

249. Zhu, F., et al., *Targeted engineering and scale up of lycopene overproduction in Escherichia coli.* Process Biochemistry, 2015. **50**: p. 341-346.

250. Huang, L., et al., *Engineering of global regulator cAMP receptor protein (CRP) in Escherichia coli for improved lycopene production.* Journal of biotechnology, 2015. **199**: p. 55-61.

251. Wu, T., et al., *Engineering membrane morphology and manipulating synthesis for increased lycopene accumulation in Escherichia coli cell factories.* 3 Biotech, 2018. **8**: p. 1-8.

252. Wei, Y., et al., *Enhanced production of biosynthesized lycopene via heterogenous MVA pathway based on chromosomal multiple position integration strategy plus plasmid systems in Escherichia coli.* Bioresource technology, 2018. **250**: p. 382-389.

253. Liu, N., et al., *Lycopene production from glucose, fatty acid and waste cooking oil by metabolically engineered Escherichia coli.* Biochemical Engineering Journal, 2020. **155**: p. 107488.

254. Kang, W., et al., *Modular enzyme assembly for enhanced cascade biocatalysis and metabolic flux.* Nature Communications, 2019. **10**.

255. Ma, T., et al., *Lipid engineering combined with systematic metabolic engineering of Saccharomyces cerevisiae for high-yield production of lycopene.* Metabolic engineering, 2019. **52**: p. 134-142.

256. Xie, W., et al., *Construction of lycopene-overproducing Saccharomyces cerevisiae by combining directed evolution and metabolic engineering.* Metabolic engineering, 2015. **30**: p. 69-78.

257. Luo, Z., et al., *Enhancing isoprenoid synthesis in Yarrowia lipolytica by expressing the isopentenol utilization pathway and modulating intracellular hydrophobicity.* Metabolic engineering, 2020.

258. Nicolás-Molina, F.E., E. Navarro, and R.M. Ruiz-Vazquez, *Lycopene over-accumulation by disruption of the negative regulator gene crgA in Mucor circinelloides.* Applied Microbiology and Biotechnology, 2008. **78**: p. 131-137.

259. Wang, G.-S., et al., *High-Level Production of the Industrial Product Lycopene by the Photosynthetic Bacterium Rhodospirillum rubrum.* Applied and Environmental Microbiology, 2012. **78**: p. 7205 - 7215.

260. Su, A., et al., *Metabolic Redesign of Rhodobacter sphaeroides for Lycopene Production.* Journal of agricultural and food chemistry, 2018. **66 23**: p. 5879-5885.

261. Zuo, Z., et al., *Engineering Haloferax mediterranei as an Efficient Platform for High Level Production of Lycopene.* Frontiers in Microbiology, 2018. **9**.

262. Bhataya, A., C. Schmidt-Dannert, and P.-C. Lee, *Metabolic engineering of Pichia pastoris X-33 for lycopene production.* Process Biochemistry, 2009. **44**: p. 1095-1102.

263. Zhang, X., et al., *Production of lycopene by metabolically engineered Pichia pastoris.* Bioscience, Biotechnology, and Biochemistry, 2019. **84**: p. 463 - 470.

264. Li, D., et al., *Engineering CrtW and CrtZ for improving biosynthesis of astaxanthin in Escherichia coli.* Chinese journal of natural medicines, 2020. **18 9**: p. 666-676.

265. Zhang, M., et al., *Improving astaxanthin production in Escherichia coli by co-utilizing CrtZ enzymes with different substrate preference.* Microbial Cell Factories, 2022. **21**.

266. Chai, F., et al., *Heterologous biosynthesis and manipulation of crocetin in Saccharomyces cerevisiae.* Microbial Cell Factories, 2017. **16**.

267. Li, X.-R., et al., *Metabolic engineering of Escherichia coli to produce zeaxanthin.* Journal of Industrial Microbiology & Biotechnology, 2015. **42**: p. 627-636.

268. Shen, H., et al., *Dynamic control of the mevalonate pathway expression for improved zeaxanthin production in Escherichia coli and comparative proteome analysis.* Metabolic engineering, 2016. **38**: p. 180-190.

269. Liang, J., J.C. Ning, and H. Zhao, *Coordinated induction of multi-gene pathways in Saccharomyces cerevisiae.* Nucleic

Acids Research, 2012. **41**: p. e54 - e54.

270. Beuttler, H., et al., *Biosynthesis of zeaxanthin in recombinant Pseudomonas putida.* Applied Microbiology and Biotechnology, 2011. **89**: p. 1137-1147.
